# Malleability of the cortical hand map following a finger nerve block

**DOI:** 10.1126/sciadv.abk2393

**Published:** 2022-04-22

**Authors:** Daan B. Wesselink, Zeena-Britt Sanders, Laura R. Edmondson, Harriet Dempsey-Jones, Paulina Kieliba, Sanne Kikkert, Andreas C. Themistocleous, Uzay Emir, Jörn Diedrichsen, Hannes P. Saal, Tamar R. Makin

**Affiliations:** 1 Institute of Cognitive Neuroscience, University College London, London, UK.; 2 Wellcome Centre for Integrative Neuroimaging, University of Oxford, Oxford, UK.; 3 Department of Neurobiology, Harvard Medical School, Boston, MA, USA.; 4 Active Touch Laboratory, Department of Psychology, The University of Sheffield, Sheffield, UK.; 5 School of Psychology, University of Queensland, Brisbane, Australia.; 6 Nuffield Department of Clinical Neurosciences, University of Oxford, Oxford, UK.; 7 Brain Function Research Group, University of the Witwatersrand, Johannesburg, South Africa.; 8 Brain and Mind Institute, University of Western Ontario, London, Canada.; 9 Wellcome Centre for Human Neuroimaging, University College London, London, UK.

## Abstract

Electrophysiological studies in monkeys show that finger amputation triggers local remapping within the deprived primary somatosensory cortex (S1). Human neuroimaging research, however, shows persistent S1 representation of the missing hand’s fingers, even decades after amputation. Here, we explore whether this apparent contradiction stems from underestimating the distributed peripheral and central representation of fingers in the hand map. Using pharmacological single-finger nerve block and 7-tesla neuroimaging, we first replicated previous accounts (electrophysiological and other) of local S1 remapping. Local blocking also triggered activity changes to nonblocked fingers across the entire hand area. Using methods exploiting interfinger representational overlap, however, we also show that the blocked finger representation remained persistent despite input loss. Computational modeling suggests that both local stability and global reorganization are driven by distributed processing underlying the topographic map, combined with homeostatic mechanisms. Our findings reveal complex interfinger representational features that play a key role in brain (re)organization, beyond (re)mapping.

## Teaser

The somatosensory representation of an anesthetized finger is preserved alongside hand-wide suppression of nonblocked fingers.

## INTRODUCTION

Representation in the primary somatosensory cortex (S1) has long been conceptualized as a somatotopic gradient, i.e., neighboring subregions are selective in their responses to neighboring parts of the body. The primate hand area is a well-established model for such somatotopic organization. Traditionally, the hand area has been divided into discrete clusters, each selective to a single finger, lined up mediolaterally—hereafter referred to as the hand map (*1*, *2*). However, underneath this apparent selectivity, finger representation in S1 is also distributed. Electrophysiological work with nonhuman primates has provided evidence for receptive fields that can not only span multiple fingers (*3*) but also cover a wider area than alluded to by mesoscale topographical mapping studies (*4*, *5*). Shared interfinger representation may be the result of input synchronization: Manfredi and colleagues (*6*) have demonstrated that even passive stimulation of a part of the hand triggers extensive ripple effects across the skin [see also (*7*)]. Because these ripples activate mechanoreceptors on other fingers, even localized tactile stimulation elicits widely distributed peripheral responses, with distinct spatiotemporal patterns (*8*). Together with related findings characterizing the organizational features of the S1 hand map that are not necessarily anchored to topographic maps (*9*–*12*), these results suggest that shared somatosensory processing of inputs from multiple skin surfaces across the hand is more prevalent than typically appreciated.

Despite these known interfinger representational motifs, the hand map has been an incredibly powerful mesoscale model for studying S1 organization and, as changes to the spatial organization of the finger clusters are relatively easy to monitor, for studying adult plasticity (*13*). Foundational literature on somatosensory plasticity, mainly in animal models, shows that the boundaries between fingers within the hand map (or whiskers in the S1 barrel cortex) are profoundly altered following localized input loss, both at very short time frames of minutes/hours (*14*, *15*) and long-term time scales (see further discussion below) (*16*, *17*). These studies demonstrated that deprived cortical territory can be activated by inputs from cortically neighboring body parts. Famously, following amputation of fingers in new-world monkeys (e.g., D3), Merzenich *et al*. (*18*) described an invasion of the neighboring fingers (e.g., D2 and D4) into the deprived area 3b cortical territory [see (*19*) for comparable findings in area 1]. Multiple processes have been suggested to drive such marked remapping, including anatomical changes (*20*). However, consensus in the field now favors the unmasking of previously silent inputs as a key process (*21*–*23*), which could even occur on a time scale of minutes (*24*). Subtle alterations in selectivity profiles outside the deprived cortex (i.e., across the hand map) were already observed by Merzenich *et al*. (*14*), but the vast majority of studies have focused on characterizing reorganization locally, within the deprived cortex, leaving more global plasticity relatively unexplored.

The findings described above suggesting widespread reorganization are seemingly contradictory with evidence from functional magnetic resonance imaging (fMRI), transcranial magnetic stimulation (TMS), and peripheral intracortical stimulation techniques in humans that suggest a persistent functional representation of missing body parts after amputation [reviewed in (*25*)] and deafferentation (*26*, *27*). We (*28*, *29*) and others (*30*, *31*) have shown that, even decades after amputation of a hand, phantom hand movements evoke activity patterns in S1 that are largely indistinguishable from normal hand movement. Together, this indicates that the canonical features of the hand representation are preserved within the sensorimotor system, not only in the short-term but also after long-term input loss.

Although these studies suggesting that representations are maintained differ from the classic reorganization work discussed above in experimental species, acquisition techniques, extent of cortex studied, and manner of stimulation (mainly passive stimulation versus active movement), the goal of both groups of studies tends to be to extrapolate to mesoscale population-level organization that underlies the hand (or body) S1 map. At that level, the dominant (but not universal) interpretation of the classic work personified by Merzenich that the deprived cortex is rapidly overwritten clashes with persistent hand representation in humans. We hypothesized that this apparent contradiction may have resulted from a strong focus on selectivity as a key organizing principle in S1, at the expense of the known overlap across finger representations [see also (*12*)]. At least in the short term, what appears to be local remapping of input to the deprived cortex does not have to be caused by the reorganization of sensory cortex.

**Fig. 1.**
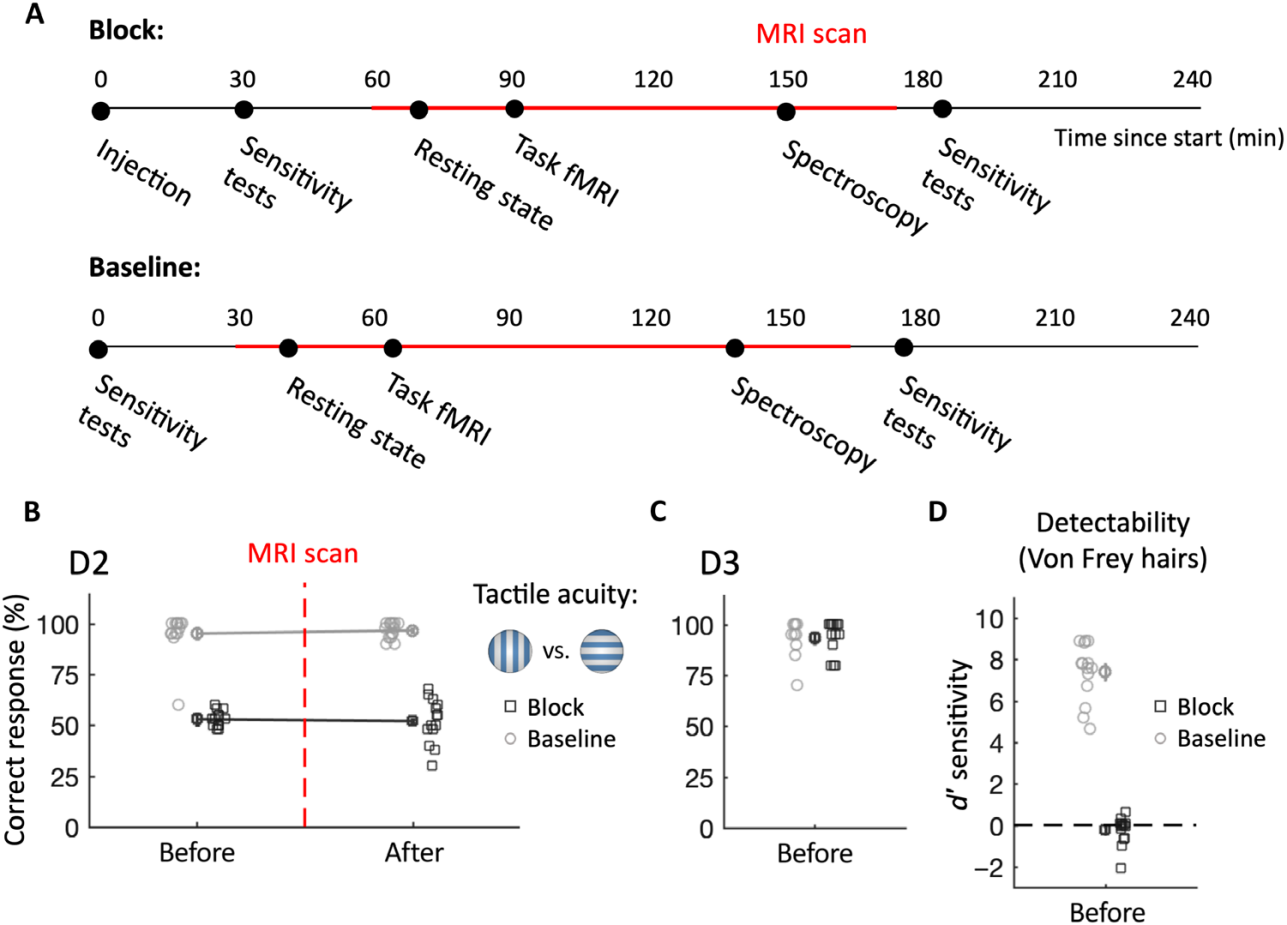
Successful administration of a local nerve block. (**A**) Experimental timeline. Dots indicate the approximate start of each study component. Time spent in the MRI scanner is marked in red. Both sessions were identical except for the injection that was only administered in the block session and the independent localizer task that was only performed in the baseline session. (**B**) Tactile acuity, assessed using a grating orientation judgment task, is abolished for the index finger (D2) following a local nerve block. During the baseline session, participants had near-ceiling performance. Performance remained stable throughout each experimental session. The (categorical) *x* axis is jittered for visualization purposes. (**C**) Tactile acuity was unaffected in the nondeprived and neighboring middle finger (D3), confirming that our intervention was localized to D2. (**D**) Detectability of light touch in finger D2 drops to chance level following nerve block.

In this study, we mimicked the rapid cessation of somatosensory input typically associated with amputation, using a pharmacological nerve block, allowing us to longitudinally characterize deprivation-triggered changes to the human S1 hand map (for previous studies using pharmacological manipulations to study deprivation-triggered remapping, see Discussion). Healthy volunteers underwent two sessions of hand mapping experiments using ultrahigh-field fMRI (1-mm resolution), one while their right index finger was locally blocked using a pharmacological anesthetic agent (block session) and another without a block (baseline session; see Fig. 1A for experimental timeline). To modulate the level of afferent and efferent input, we probed S1 with both passive (tactile stimulation) and active (finger tapping) stimulation [see Sanders *et al*. (*32*) for a comparison between active/passive conditions on S1 hand representation at baseline]. We hypothesized that peripheral and central processing being shared across fingers would cause the blocked finger’s “missing” activity to be reinstated by the other fingers, leading to maintained representation. With regards to classical accounts of deprivation-triggered remapping, we predicted that, if fingers are more overlapping across the hand map to begin with, then, simply by removing the dominant input from a given finger cluster, we will expose the neighboring fingers’ representation. Unless an additional plasticity mechanism was involved, which we believed would not play a large role at this time scale, deprivation in itself should not boost the activity for the neighboring nonblocked fingers. By contrast, because the same neurons underlie multiple fingers’ representations, we hypothesized that reduced input to one anesthetized finger would decrease activity for the other (nonblocked) fingers.

## RESULTS

The use of active and passive tasks was originally aimed at resolving some possible causes of methodological divergence in human and animal studies. However, given the overall consistency in the results between the active and passive conditions, the analyses reported below focus on the passive condition; parallel results from the active condition are reported in the Supplementary Materials. We highlight instances where results diverged between the two paradigms.

### Local nerve block attenuates tactile acuity and local activity

We first established that perception for the index finger (D2) was effectively attenuated in the block session using a tactile grating orientation judgment test. All participants showed near-ceiling performance in the baseline session (98%; see Fig. 1B), whereas performance dropped to chance level after the nerve block (53%). Performance remained poor after the end of the scan, 3 hours after the block was administered [main effect of session: χ^2^_(1)_ = 1358, *P* < 0.001; time × session interaction: χ^2^_(2)_ = 0.12, *P* = 0.729]. Before the scan (and after the nerve block in the block session), we also tested for reductions in perception of light touch, the slowest somatosensory modality to be abated by a nerve block (*33*), using Von Frey hairs (Fig. 1D). Participants’ sensitivity to detect light touch was reduced from nearly perfect to chance level after the nerve block [χ^2^_(1)_ = 324.73, *P* < 0.001]. We also tested the acuity of the neighboring middle finger to ensure that the effects of the nerve block had not spread to other fingers. We noted near-ceiling grating orientation performance in both sessions before the scan [and after the block in the block session; baseline: 92%; block: 93%; main effect of session: χ^2^_(1)_ = 0.26, *P* = 0.609; Fig. 1C]. At the end of each session, we directly assessed the participants’ perceptual thresholds for the middle (D3) and ring finger (D4) using gratings (see Methods for details). There was no significant main effect of session [χ^2^_(1)_ = 1.03, *P* = 0.311] nor a finger × session interaction [χ^2^_(2)_ = 0.37, *P* = 0.541], further supporting that the effect of the nerve block had not spread to the nontarget fingers.

**Fig. 2.**
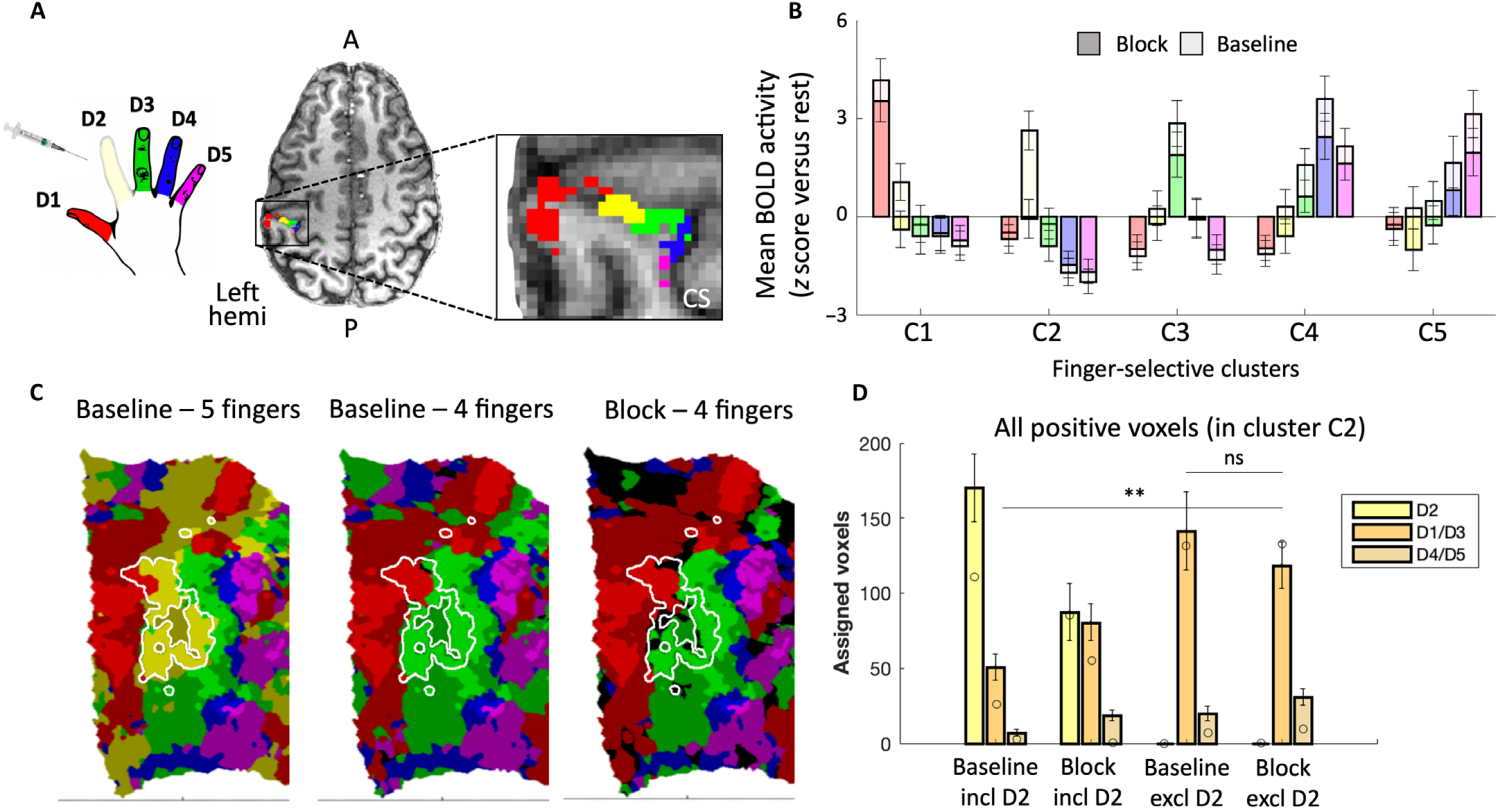
Single-finger nerve block attenuates D2 activity but does not cause true reorganization—evidence from univariate analysis. (**A**) An example of the five finger clusters that were localized in S1 for each participant. CS, central sulcus. (**B**) Activity levels for each of the five fingers [colors as in (A)] across the five finger clusters in the baseline (light colors) and block (dark colors) sessions. Group results for passive stimulation. In the block session, activity for finger D2 is abolished in cluster D2 but also decreases for other target fingers in their respective clusters. Error bars indicate SEM. BOLD, blood oxygen level–dependent. (**C**) Winner-takes-all assignment of voxels to fingers, presented on a flattened surface for an example participant, for positive (above-zero) activity only [colors as in (A)]. The white outline shows the D2-selective cluster, as identified independently. Compared to measuring five fingers in the baseline sessions (left), not measuring finger D2 reveals similar “invasion” of the neighboring fingers (D1, red; D3, green) into the D2 territory (white outline), irrespective of a nerve block, resulting in similar “remapping” in baseline as found in the block map. Bright and shaded colors indicate activated (*Z* > 0) voxels inside and outside the independently localized finger clusters, respectively; black indicates below-zero activity. (**D**) Quantification of the remapping effect demonstrated in (C). Group results. Voxels within the D2 cluster were assigned to either one of five fingers (including D2) or one of four fingers (excluding D2) using a winner-takes-all approach. The number of voxels assigned to D2 (when included), neighboring fingers (D1/D3), and non-neighboring fingers (D4/D5) were calculated per participant and averaged. Error bars indicate SE. Circles indicate the values for the example participant that was showcased in (C). A, anterior; P, posterior; Left hemi, left hemisphere; ns, nonsignificant. **P < 0.001.

We next demonstrated that the nerve block was physiologically effective in diminishing D2 activity in its respective S1 area. Considering that S1 is not homogeneous in finger selectivity, we first examined the S1 regions showing the greatest selectivity to one finger over the other four. The five “finger clusters” (C1 to C5) were identified in each participant’s hand area by means of a standard traveling wave analysis performed on an independent localizer map (Fig. 2A; see Methods). While we could not accurately isolate the subdivisions of S1 in individual participants, on the basis of probabilistic mapping, we did confirm that these finger clusters were most likely found in the more finely tuned areas 1 and 3b (see Methods). Within each finger cluster, mean finger-specific activity levels were obtained from the randomized block design task collected during both baseline and block sessions (Fig. 2B). As expected, after the nerve block, passive stimulation of D2 no longer induced activity in its corresponding finger cluster (C2) relative to rest (μ = −0.04; *t*_14_ = −0.10, *P* = 0.919, Bayes’ factor = 0.266; decrease versus baseline session; *t*_14_ = −4.17, *P* = 0.001). Active D2 stimulation still elicited positive activity in C2 (μ = 2.13; *t*_14_ = 4.40, *P* = 0.001; see fig. S1A), presumably because of additional afferent (e.g., proprioceptive inputs from the arm muscles) and efferent signals (from the motor system). Note however that, in the active condition, the activity was also significantly reduced in the block condition relative to baseline (*t*_14_ = −2.22, *P* = 0.044).

Activity elicited by (passive) stimulation of D2 was also reduced in the other four finger-selective clusters compared to baseline (*F*_1,112_ = 9.90, *P* = 0.002), with no significant interaction across clusters (*F*_3,112_ = 0.48, *P* = 0.700). Thus, the nerve block successfully attenuated D2 univariate activity across the hand area.

### Local “remapping” might reflect analysis choices rather than neural plasticity

Classical electrophysiological studies involving amputation have reported an overrepresentation of neighboring fingers in the deprived territory. To use methods similar to those studies, we first calculated winner-takes-all maps (Fig. 2C), rather than using the full activity patterns, across the entire S1 hand area. In these maps, all voxels showing positive (above zero) activity to one of the fingers were assigned to the finger with the highest activity level (relative to baseline). Similar findings were observed when activity was not thresholded, such that negative blood oxygen level–dependent (BOLD) activity was also considered (see fig. S2).

The finger preference of the voxels in the highly selective cluster C2 was measured by counting the number of voxels assigned to the neighboring fingers. We first repeated the classical analysis, ignoring the D2 condition in the winner-takes-all map of the block session—a necessity in animal studies involving amputation—but not in the baseline session. When comparing the number of assigned voxels across sessions, we find a significant increase in the number of voxels that show a preference for D1 or D3 after a nerve block, compared to the baseline session (*t*_14_ = 5.10, *P* < 0.001; see Fig. 2C for an example map and Fig. 2D for group comparisons). This is consistent with previous accounts of local remapping caused by overrepresentation of neighboring fingers (see Introduction). However, a key advantage to our experimental model is the possibility to stimulate D2 after deprivation. When D2 responses are included in the winner-takes-all analysis, the remapping of neighboring fingers D1 and D3 in C2 is merely trending (*t*_14_ = 2.13, *P* = 0.051). Furthermore, ignoring D2 in the baseline data already gives the impression of shifted finger boundaries (four fingers winner-takes-all relative to the five fingers comparison, both at baseline; *t*_14_ = 4.84, *P* < 0.001; Fig. 2D). Conversely, when D2 was ignored in both sessions (i.e., excluded from the winner-takes-all analysis), we found no significant effect of the nerve block on neighboring fingers’ remapping in the deprived cluster (*t*_14_ = −1.12, *P* = 0.280). This suggests that the previously observed findings, which we were able to replicate within the highly selective C2 cluster, might have been the product of methodological restrictions. In other words, remapping or other mechanisms of plasticity may not be necessary for explaining a change in the winner-takes-all maps.

The conclusion that remapping of the cortical neighbors into the deprived cortex may not have occurred in our study was further supported by other analysis, focusing on the mean activity within the finger clusters. The mean activity in C2 for fingers D1 and D3 did not significantly increase in the passive condition (*t*_14_ = −1.09, *P* = 0.304; Fig. 2B) and had, in fact, decreased in the active condition (*t*_14_ = −2.51, *P* = 0.025). Together, these results suggest that the changes in the hand map came about by uncovering preexisting coactivation of voxels by fingers other than the “winning” (target) finger.

### Global loss of selectivity in the hand region

Given the hypothesized overlap in finger representation, we also investigated the possibility for more global changes in the hand representation, i.e., outside the deprived area, but still within the subdivision, showing high selectivity for individual fingers. We first examined whether responses within the finger clusters beyond C2 were affected by the nerve block by assessing univariate finger selectivity: the activity for each cluster’s target finger minus the mean of the three nontarget fingers, excluding D2 (see the Supplementary Materials for the same analysis while including D2). Finger selectivity is considered a hallmark of sensory cortical organization, particularly in S1. With passive stimulation, we identified a significant decrease in selectivity across all (nonblocked) finger clusters (mean change: −21.7%; *F*_1,112_ = 8.14, *P* = 0.005), and no session × cluster interaction (*F*_3,112_ = 0.31, *P* = 0.818). This result provides clear evidence for larger-scale changes in activity across the highly selective finger clusters, which have not previously been emphasized. When examining Fig. 2B, it is apparent that the nontarget fingers are not equally suppressed within a given cluster, but vary in a systematic fashion, in line with topography. That is, neighboring fingers that were more strongly activated at baseline also show greater suppression (because of floor and ceiling effects, this observation could not be tested statistically).

**Fig. 3.**
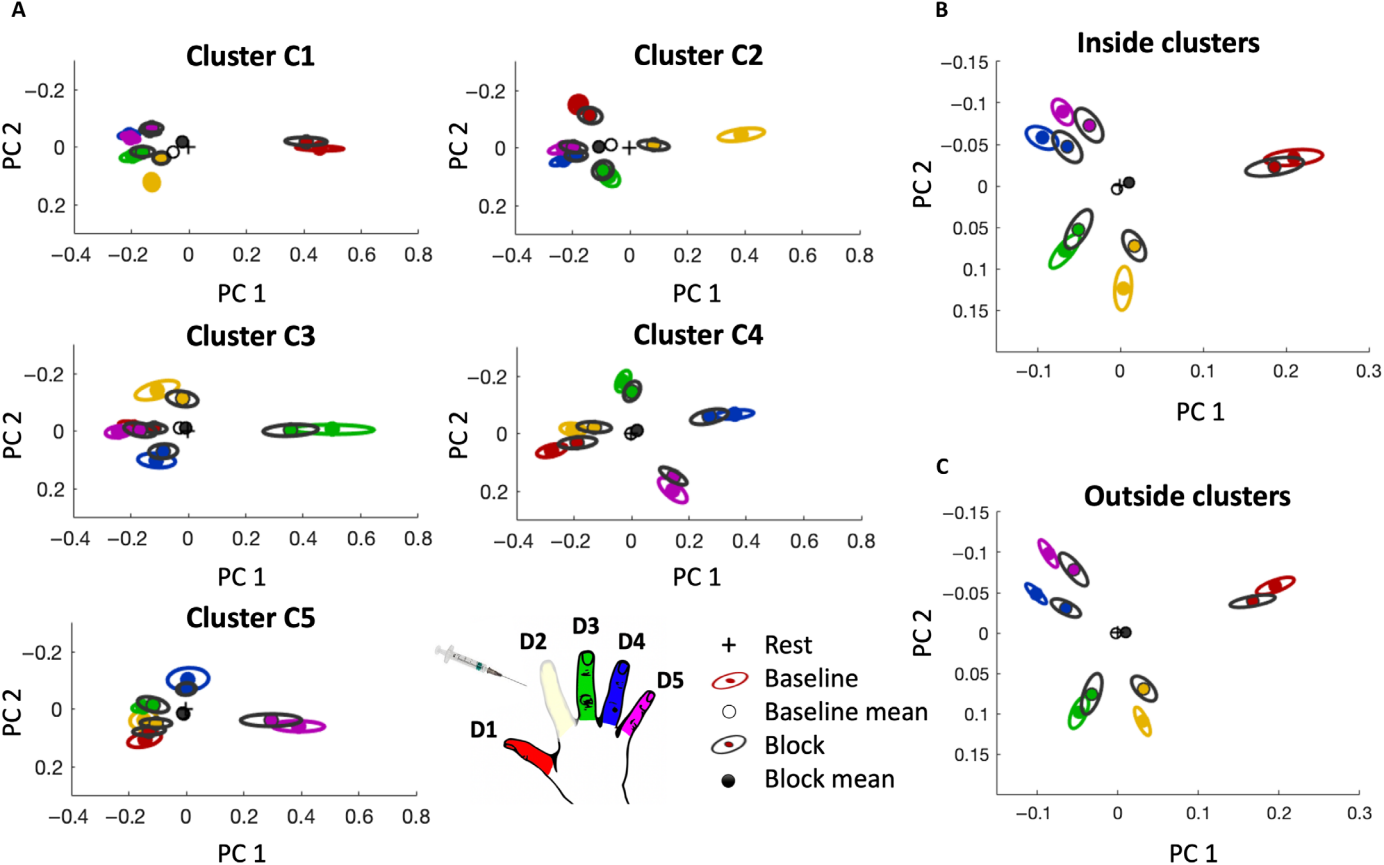
Finger representation is distributed throughout the hand area and globally affected by a local nerve block. (**A**) In each finger cluster (C1 to C5), both target and nontarget fingers show differential activity patterns from each other and from rest. Fingers are colored according to the hand-shaped legend; the surrounding ellipses (indicating between-subject SE) are either colored (baseline session) or black (block session). The interfinger representational structure (composed of all five fingers) is relatively consistent across sessions. (**B**) When all clusters are examined collectively, the prototypical hand representation shrinks following the nerve block but does not change shape. (**C**) Hand representation excluding the finger-selective clusters C1 to C5 strongly resembles that of the highly selective voxels, suggesting that hand information is distributed throughout the S1 hand area. PC, principal component.

To investigate the global selectivity reduction in more detail, we conducted representational similarity analysis (RSA; see Methods). RSA produces a canonical S1 representational structure for “normal” hands based on interfinger similarity patterns and can therefore be used to assess variations in interfinger dissimilarity. While extensively previously used to characterize the fingers’ representational structure across the entire hand area, here, we used the same approach to identify representational similarity within each finger-selective cluster. For each cluster, the dissimilarity between all individual finger patterns, as well as rest, was compared in each session. For visualization purposes, the representation patterns were projected onto a two-dimensional plane using multidimensional scaling (MDS) (Fig. 3A), such that distances in the projection reflect dissimilarity between conditions [for representational dissimilarity matrices (RDMs), see fig. S3]. Given that we used cross-validated distances (see Methods), systematically positive differences imply statistically reliable differences between patterns.

We first consider the interfinger representational structure in each of the finger-selective clusters in the baseline session. Selectivity of each of the finger clusters as defined above can be inferred with RSA by the target finger showing the highest dissimilarity from rest, relative to the nontarget fingers (Fig. 3A, colors). The nontarget fingers in each of the five clusters, however, can also be distinguished from each other (mean dissimilarity between nontarget finger pairs per cluster; *t*_12–14_ > 5.90, *P* < 0.001; one-sample *t* tests). In three of five clusters, the dissimilarity distance rank between nontarget fingers (e.g., D3 is closer to the D4 than to D5) was preserved (average rank-order correlation with the canonical hand pattern, i.e., the pattern from the entire hand area in the baseline session: C1: rho = 0.64, *P* = 0.038; C2: rho = 0.55, *P* = 0.024; C3: rho = 0.26, *P* = 0.083; C4: rho = 0.46, *P* = 0.122; and C5: rho = 0.51, *P* = 0.008). This analysis suggests that, at baseline, finger information is highly distributed, such that even the most finger-selective clusters contain whole-hand topographic information.

We next interrogated the impact of the D2 nerve block on the S1 hand representational structure beyond the highly selective clusters, in an anatomically defined region around the “hand knob” spanning the entire hand area (see Methods; further analysis on the impact of the nerve block on the representational structure in the finger-selective clusters is elaborated below). To avoid overlap with the observations presented above, we excluded all voxels with pronounced selectivity for a single finger, i.e., voxels inside finger clusters. Akin to the reduction in finger selectivity in Fig. 2, interfinger dissimilarity due to passive stimulation was significantly reduced in the extended hand area across the four nonblocked fingers following the nerve block (−29%, *t*_13_ = 3.00, *P* = 0.010). Neighboring fingers (D1 and D3) were not affected to a significantly different extent than non-neighbors (D4 and D5; finger neighborhood × session interaction: *F*_1,52_ = 0.12, *P* = 0.731). Similar results were also observed when only the activity underlying the finger-selective clusters was examined (−26%, *t*_13_ = 2.54, *P* = 0.025; Fig. 3B). As can be seen by comparing Fig. 3 (B and C), the representational structure in the most finger-selective voxels and the nonselective voxels comprising the anatomical hand area is highly similar (mean within-subject correlation: baseline session: *r* = 0.88; block session: *r* = 0.89). This means that, despite the known varied scope for interfinger overlap across the different cytoarchitectonic division of S1 and specifically within and outside the highly selective clusters, the representational structure of the hand was similarly affected by the D2 block.

There was a similar drop in dissimilarity during the active task (inside clusters: −19%, *t*_14_ = 3.45, *P* = 0.004; outside clusters: −21%, *t*_14_ = 4.40, *P* = 0.001; fig. S1), also without a significant finger neighborhood × session interaction (*F*_1,56_ = 0.20, *P* = 0.660). The measured drop in dissimilarity could not, to the best of our knowledge, be attributed to intersession differences that are unrelated to the nerve block, e.g., task performance or brain state. We determined that there were no significant differences between sessions in press force during (active) task performance (*t*_14_ = −0.81, *P* = 0.433) or perceptual detection of an oddball trial during (passive) task performance (*t*_14_ = 0.34, *P* = 0.737), the residual error in the general linear model (GLM) (*t*_14_ = 0.86, *P* = 0.405), or the signal variance in S1 in a separate resting-state scan (*t*_12_ = 0.40, *P* = 0.700; see Methods). Collectively, the above findings provide converging evidence that individuated finger representation became less distinct across the hand area following D2 block. Although the deprivation of input was local, i.e., affecting only one finger, finger information is distributed, leading to widespread effects on the representation of the nonblocked fingers both within other finger-selective clusters and beyond.

### Persistent representation of the blocked finger is found outside the C2 cluster

From our analysis so far, it becomes apparent that the reduction of D2 activity via nerve block affected the nonblocked fingers’ representation across the hand area. We next explored the idea that, because of distributed and overlapping finger representation, activity associated with one finger may inform the representation of another finger or could even reinstate it in the case of missing input (e.g., D2 representation in the block session). Going back to the (passive) cluster-specific analysis in Fig. 3A, the blocked D2 representation is significantly different from the rest condition in all highly selective finger clusters (*t*_13_ > 3.87, *P* < 0.002). Looking at the specific effects of the D2 block, it appears that, while D2 dissimilarity drops sharply compared to rest in cluster C2, in the other clusters, it is more stable. Statistically, we identified a significant dissimilarity drop in clusters C1 (*t*_13_ = 2.50, *P* = 0.027) and C4 (*t*_13_ = 2.43, *P* = 0.031) but not in clusters C3 (*t*_13_ = 1.09, *P* = 0.297) and C5 (*t*_13_ = 1.21, *P* = 0.247). As exemplified in the hand area analysis detailed above, this dissimilarity drop is not specific to D2. When each cluster’s four nontarget fingers are examined compared to rest, no cluster shows a significant session × finger interaction (0.11 < *F*_3,104_ < 0.81; *P* > 0.489), yet every cluster showed a main effect of session (*F*_1,104_‘s > 7.73; *P*’s < 0.008). In other words, while we see a general decrease in finger-specific information, this decrease seems relatively stable across the nonblocked fingers. The global dissimilarity reduction across the nonblocked fingers was also evident when the fingers were grouped as neighbors and non-neighbors to the target finger (effect of session: *F*_1,108_ > 6.90, *P* < 0.010; session × neighborhood interaction: 0.00 < *F*_1,108_ < 1.64, *P* > 0.204). These findings are in contrast to the idea of increased selectivity of the cortical neighbors due to local cortical remapping.

To test whether the blocked D2 sustained greater reduction in representation relative to the other fingers, we examined the entire hand area (excluding the finger-selective clusters; Fig. 3C). We again do not find significant evidence for a greater collapse in dissimilarity from rest for any of the fingers (including all five fingers; effect of session: *F*_1,130_ = 5.99, *P* = 0.002; session × finger interaction: *F*_4,130_ = 0.07, *P* = 0.991). Therefore, the reduction in information about D2 as a consequence of the nerve block was, with the exception of cluster C2, not notably different from that about the nonblocked fingers. In other words, while locally, representation was abolished in the highly selective C2 cluster, in the greater hand area, the localized nerve block triggered a relatively homogeneous reduction in dissimilarity across fingers.

In the active condition, no cluster showed a significant session × finger interaction (0.20 < *F*_3,112_ < 1.12, *P* > 0.344). Only C4 showed a significant main effect of session (C4: *F*_1,112_ = 18.46, *P* < 0.001; other clusters: 0.15 < *F*_1,112_ < 3.07, *P* > 0.083). Active results from the entire hand area (excluding the finger-selective clusters) are in line with that of the passive condition (including all five fingers; effect of session: *F*_1,140_ = 10.7, *P* = 0.001; session × finger interaction: *F*_4,140_ = 0.21, *P* = 0.930). Together, this suggests that, despite the reported reduction in D2 activity across the hand map, the finger’s position within the canonical hand representational structure is preserved.

### Homeostatic mechanisms can support global representational stability

**Fig. 4.**
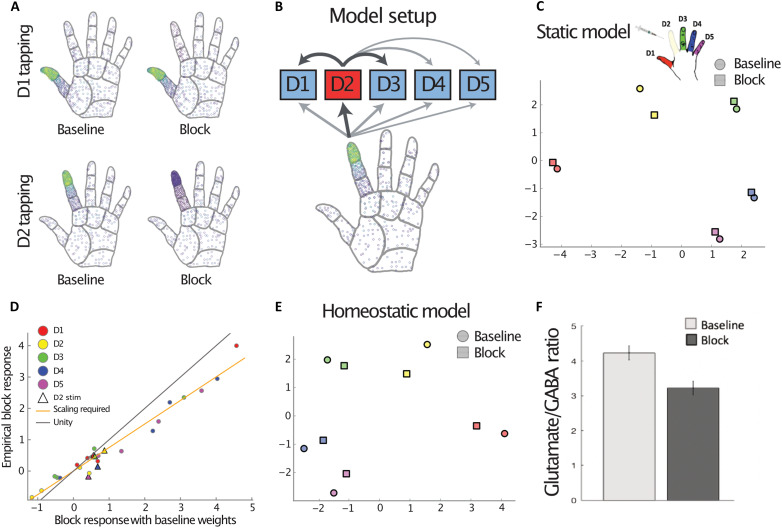
A computational model implementing a combination of local peripheral block and homeostatic mechanisms can account for the observed results. (**A**) Simulated peripheral responses to mechanical finger stimulation (akin to the one used in the passive condition) in the baseline and block sessions, with lighter colors indicating higher firing rates. (**B**) The cortical model consists of five units representing the different finger-selective clusters, which receive input from the periphery and are laterally connected. Thicker arrows denote stronger connections. (**C**) A model fit on baseline responses based on simulated peripheral inputs cannot account for the observed results under peripheral D2 nerve block. (**D**) Predicted univariate activity under D2 block for the static model (i.e., with baseline weights) against empirically observed results (see Fig. 2B). Most activity is well fitted by a constant gain decrease (orange line fit to circles), but a separate mechanism is needed to account for activations arising from the blocked D2 stimulation condition (triangles). (**E**) A model incorporating a global gain decrease and a selective D2 increase can reproduce the observed empirical pattern (see Fig. 3). (**F**) A significantly decreased glutamate/GABA ratio following the block, as identified using MRS in the cortical hand area. The reduction in excitation/inhibition could be an empirical in vivo marker of the global gain decrease required by our model.

Going back to the idea that even localized stimulation causes peripheral ripples across the hand (Fig. 4A), it is possible that the findings described thus far are entirely driven by the peripheral effects incurred by the nerve block. According to this framework, if D2 inputs normally contribute to cortical representation of all other fingers, then it stands to reason that blocking D2 will reduce activity profiles of all other fingers. Similarly, if the cortical D2 representation is partially constructed by peripheral inputs from the other fingers, then these inputs should be enough to sustain the D2 representation despite the block. To disambiguate whether the observed activity changes in the block condition can be attributed to the loss of peripheral input alone or whether additional central plasticity mechanisms are at play, we used a computational model that simulates S1 hand representation based on peripheral inputs (see Methods for details). In short, this model makes explicit that passive finger stimulation causes ripples in the skin that can activate mechanoreceptors beyond the immediate stimulation site (*7*). The cortex is modeled with five units (representing the five cortical finger-selective clusters) that receive input from the periphery and are also connected laterally (Fig. 4B). Including broad interconnectivity between different cortical patches allowed the model to encapsulate the canonical two-dimensional hand representation in S1, as found using RSA in this and previous studies (*9*, *34*, *35*).

We first tested whether the loss of peripheral input on its own might account for the observed cortical changes. After fitting the model to the empirical data obtained in the baseline session (see Methods), we examined the effects of the peripheral nerve block on the cortical representations, assuming that cortical response profiles did not change. As mechanical stimulation of a single finger excites tactile afferents terminating on other fingers and the palm, the loss of peripheral input from one finger could, in theory, affect other cortical patches. However, we found that the simulated activity pattern under anesthesia differed from the observed one, with the static model predicting a greater collapsing of D2 representation, relative to the other fingers (square markers in Fig. 4C). This simulation suggests that other central mechanisms likely mediate the observed effects of peripheral input loss, in addition to the distributed peripheral input.

To further explore potential central changes, we compared the simulated univariate activity under the static model with the empirically observed activity. This revealed that the cortical changes were driven by two simple effects. First, a global reduction in gain by approximately 25%, such that activity was reduced proportionally for all cortical units under all stimulation conditions (see fitted slope versus unity line in Fig. 4D), which accounted for most of the block effect other than when D2 was stimulated. Second, the D2-specific maintained representation could be explained by a gain change in the D2 unit that increased its activity by approximately 25% and propagated through lateral connection to other cortical clusters (see initial model illustration in Fig. 4B). Both effects together reproduced the observed cortical changes in the block condition (Fig. 4E). The proposed gain changes are consistent with homeostatic mechanisms: Decreased activity in the D2 unit might have elevated D2’s gain to raise this cluster’s activity, simultaneously with globally decreased gain.

Preliminary physiological evidence collected using MR spectroscopy (hereafter MRS) provides some support of the mechanisms behind the global gain changes proposed by the model. Enhanced excitability specific to the D2 patch may be difficult to show given the resolution of our methods. The widespread excitability drop for the other fingers, however, was empirically supported by changes in the neurochemical profile of S1. In a subset of participants (*n* = 10), we successfully performed MRS over the (entire) sensorimotor hand area in both baseline and block sessions. The glutamate/γ-aminobutyric acid (GABA) ratio at rest, a marker of cortical excitability (*36*), was reduced in the S1 hand area after block (*t*_9_ = 2.48, *P* = 0.035; Fig. 4F). Although further research is warranted, this global reduction in excitation/inhibition strength provides support for a nonspecific homeostatic plasticity mechanism that can realistically take place at the rapid time scale that applies here.

## DISCUSSION

Textbook electrophysiological findings suggest that neighboring fingers will invade the cortical territory of a silenced finger, and this remapping of boundaries within the hand map is often taken as evidence for reorganization. In our analysis, we followed the winner-takes-all method used in previous studies to show local remapping. Consequently, we were able to reproduce this classical result: The “deprived” D2 territory was “overtaken” by the neighboring D1 and D3 representations (Fig. 2C). We emphasize that, while originally observed in the monkey in areas 3b/1 and following chronic deafferentation/amputation (*18*, *19*), similar local remapping results have since been found in various animal models, from rodents (*15*) to humans (*37*); over multiple time frames, from minutes (*24*) to decades (*16*); and using an incredibly wide range of methodologies, from optical imaging (*38*) to electrical source imaging using magnetoencephalography (MEG) (*37*). Therefore, while our methodology was distinct from the original electrophysiological studies performed in the 80s on multiple accounts, there is an abundance of related reports on local remapping that bridge the gap. Several of the above researchers attributed their findings to rapid unmasking, i.e., the desilencing of intact but dormant neural connections from neighboring fingers, whereas we also consider overlap with nondominant finger representations already active before input loss. The principal distinction between our study and many previous studies of local remapping is that we have aimed to set our replication against the hypothesis that such interfinger representational overlap hinders representational change, motivating us to further characterize the interfinger representational features underlying the deprived hand map.

We demonstrate that despite apparent remapping, in our dataset (based on short-term deprivation), there is little evidence that voxels at baseline selective to the blocked finger increase their preference for that finger’s neighbors following deprivation. Instead, we identified a significant reduction in finger activity and selectivity that extended well beyond the deprived finger cluster and was evenly spread across both neighboring and non-neighboring fingers. Furthermore, despite a drastic reduction in activity for the blocked finger in voxels that were highly selective to that finger, we found clear evidence for that finger’s persistent representation elsewhere in the hand area. This result is consistent with recent findings, showing a preserved interfinger representational structure in chronic amputees (*29*). Our present findings showing global reduction of finger selectivity across the entire hand area are also consistent with our previous behavioral findings, where we administered a localized nerve block to one finger (D2) while training participants to improve the perceptual acuity of a neighboring finger (D3) (*39*). We found that, relative to training with a sham block, the nerve block increased the spread of learning from the trained finger to untrained fingers across the hands (i.e., even in fingers not traditionally topographically related to the trained finger, such as D4 of the opposite hand). In addition, training gains were also recorded in the blocked finger itself. These behavioral findings suggest that, consistent with our present findings, local blocking to one finger increases (the overlap of) interfinger processing.

### Why was finger selectivity reduced across the hand map?

Our first finding is that finger selectivity, characterized using both local differences in net activity and multivariate RSA, was reduced, in both the passive and active task. This goes against the intuition that local unmasking (within cluster C2) and disinhibition within neighboring clusters (due to reduced lateral inhibition) would cause increased selectivity, particularly for the neighboring fingers, even at the short time scale examined here. However, as illustrated by our computational model, when accounting for complex interfinger receptive fields (as will be discussed below), attenuating D2 input should not cause larger inhibition to clusters C1 and C3 than to clusters selective to non-neighboring fingers. The reduction in selectivity could not be accounted for solely by the reduced input from the blocked finger. Instead, as indicated by the computational model, global inhibition across the map is required to explain the observed reduction in net activity. Tonic inhibition, mediated through extrasynaptic GABA receptors, could account for this global selectivity reduction. By shifting the relative spiking threshold, receptive fields will modulate their own selectivity. Xing and Gerstein (*40*) showed that rapid receptive field change following input loss can be successfully modeled using only tonic inhibition. Our observation that blocking one finger can cause shifts across the whole hand map is consistent with the original observations by Merzenich *et al*. (*14*), who noted that local deprivation triggered more global changes to the boundaries within the hand map. This finding suggests that this process is somatotopically less precise than previously assumed: The entire hand representation undergoes inhibition. This proposed mechanism fits with the decreased glutamate/GABA ratio that we have found at rest using MRS, which also suggests an increase in inhibition after input loss.

Alternatively, it could be argued that local remapping should result in increased overlap in the receptive field properties of different fingers, resulting in increased representational similarity and decreased selectivity (as we show here). It is worth highlighting that the decreased selectivity (and increased representational similarity) was homogeneous across fingers and was found both within and outside the highly finger-selective clusters. Hence, the observed findings cannot be solely driven by local changes to receptive field properties within the deprived cortex.

We recently identified a similar whole-hand drop in selectivity, similar to what was observed here, after (able-bodied) participants adapted their hand dynamics to use an additional artificial thumb [(*35*); see Ogawa *et al*. (*41*) for similar findings in trained pianists]. Although plausible alternative theories exist for that finding [see (*35*)], reduced selectivity may occur more generally following atypical sensorimotor input. The mechanism behind our results may also be involved in other (rapid) representational shifts following changes in finger input, e.g., tonic inhibition following sustained tactile stimulation (*42*). We therefore conclude that the reduced finger selectivity observed here was aided by central plasticity mechanisms. It is important to note that, although we have hypothesized that homeostasis is achieved through a cortical mechanism, plasticity mechanisms occurring subcortically (*43*) will also influence the primary somatosensory cortex. While we are unable to comment on the relative contribution of noncortical mechanisms on the observed results, the altered inhibition-excitation ratio in S1 does suggest some role for that area in this process.

### Why was the blocked finger’s representation persistent?

The observation of persistent representation of the blocked finger is unexpected if one would consider the hand map as a mosaic of discrete finger clusters. However, as highlighted in the introduction, this is a specious argument. In the current study, while the peripheral signals leading to the hand map mainly come from mechanoreceptors on the blocked finger (in natural contexts), some peripheral receptive fields are very large, integrating information from across most of the hand [(*5*); even before reaching cortex (*44*)]. This likely arises from the mechanical attributes of the hand, which induces a ripple of vibration following even very localized touch, which will, in turn, cause a specific spatiotemporal pattern of mechanoreceptor activity across the entire hand (see also Fig. 4A) (*6*, *8*). At the cortical level, receptive fields can be complex and integrate information across a variety of receptor types (*5*, *45*). Hence, somatosensory input should be regarded as inherently distributed across the hand (and beyond), a view many have argued for before [e.g., (*9*)]. This cortical interfinger representation may also be influenced by natural hand use, where interfinger co-use is commonplace (*46*) and the fingers engage in highly regular patterns of comovement. Distributed interfinger activity can even be induced by localized microstimulation of individual mechanoreceptive afferent units (*47*), suggesting that this is an organizational feature of S1. It is thus likely that periphery-driven interfinger interactions, not readily inferred from the finger maps, are robust throughout the somatosensory system.

However, the persistent response for the blocked finger in our study was greater than what might have been expected from stimulation of the unblocked fingers’ mechanoreceptors during blocked finger stimulation. Our model suggests that remaining D2 information (from whichever source, see below) needs to be selectively boosted to produce the observed activity patterns. In other words, the global inhibition needs to be offset with respect to residual inputs relating to D2. This could possibly be driven by a homeostatic mechanism, keeping the firing rates in the deprived cortex stable. Although homeostatic plasticity is typically studied at longer time scales (*48*), it may also occur at the scale of hours and less [(*49*); see (*50*) for short-term monocular deprivation], making it a potential mechanism for the changes seen here.

A more trivial explanation for the persistent D2 representation may be that we did not perform a successful nerve block. Both the diminished univariate activity of D2 in its related S1 cluster and our behavioral results showing D2 acuity at floor level indicate that input from D2 was successfully attenuated. However, it is likely that some D2-related residual input, although reduced, still reached S1, as shown in our active condition. This is not a limitation of our pharmacological approach, but rather a shared feature with other deafferentation models for studying deprivation—even when considering extreme cases of spinal cord injury or whole-hand amputation, some rudimentary peripheral signals persist from the injured nerves (*51*). In our computational model, we assumed that the nerve block does not provide complete abolishment of all D2 inputs. While peripheral input may play a role in maintaining the representation of the amputated, deafferented, or blocked body part, even then, the magnitude of the empirically observed persistent representation exceeds what would be expected from such limited input. This is supported by our model, as well as our analyses showing that the decrease in D2 dissimilarity across the hand area was not quantitatively different from the decrease observed for the other, nonblocked, fingers.

More likely, decreased peripheral drive may have been compensated by up-regulation of residual afferent, as well as nonafferent, inputs. First, the somatosensory cortex and the motor system are tightly linked (*52*, *53*), and motor commands, unaffected by our nerve block, are also routed to S1. We have previously argued for a role for the motor system in stabilizing S1 organization following (long-term) amputation (*25*), and similar mechanisms could facilitate the persistent representation observed here. According to this interpretation, the S1 inputs contributing to the maintained representation of the deafferented finger (or limb) may be distinct from the afferent inputs comprising the “remapped” (surviving) representations [see e.g., (*54*)]. Although motor commands can explain why activity for the blocked finger was not reduced to zero during the active movement condition, any undetected micromovements during the passive task would not be expected to engage S1 strongly. Still, the global drop in selectivity was proportionally similar during both the active and passive task. Second, nonafferent inputs representing cognitive (e.g., attentional and imagery) factors have been shown to modestly modulate hand-related activity in S1 (*55*–*57*). Pattern completion using top-down input from higher-order sensory cortex has also been shown to modulate activity in the visual cortex (*58*, *59*). Because the participants were informed at all times which finger was being stimulated through visual feedback, top-down processes may have been able to fill in the blocked finger’s activity. In some ways, this is compatible with the notion of complex interfinger receptive fields, with some residual peripheral information relating to D2 stimulation (either through some residual input or aided by the rippling effect of mechanoreceptive responses following blocked D2 stimulation). Pattern completion could even be facilitated by the hypothesized horizontal connections between neurons within S1 (*60*). Crucially, here, the persistent representation will be stabilized by the “surviving” afferent representations [see (*61*) for review]. It is likely that all of these processes (both bottom-up and top-down) contribute to the persistent representation observed here. Regardless of the specific process, the key mechanism to enable the distinct representation of D2 despite abolishment of its primary input is the distributed representation across fingers, which we believe is shaped and maintained by daily life experience (*10*, *46*).

### Can methodological differences account for the divergence of findings relative to other studies?

We find widespread changes in finger selectivity following localized deprivation but, consistent with our findings in amputees, we do not observe any changes in the overall interfinger representational pattern (Fig. 3B). We do not think this result contradicts the multiple previous studies reporting remapping of cortical neighbors into the deprived sensorimotor territory (*62*). First, the short-term effects of deafferentation studied here leave the door open for substantial reorganization after more prolonged input loss. However, as highlighted above, when only considering net selectivity profiles within the deprived cortex (winner-takes-all), our data can replicate the cortical remapping shown by others. When considering additional representational facets, notably interfinger overlap, we find a shrinkage of the representational structure rather than shifted (or more distinct) representation of the neighboring fingers. Interfinger overlap and selectivity are both important aspects of the topographic hand map, and our findings provide a nuancing contrast to net selectivity alone, which has dominated human research on deprivation-triggered plasticity. This view is consistent with a recent demonstration that the information content of body parts and sub-parts is more distributed across S1 than selectivity maps suggest (*12*).

We do not find any evidence for increased activity for the neighboring fingers within the deprived cortex, as might be expected from a process of local unmasking. The time scale at which our effect occurs (within 1 to 2 hours) excludes the contribution of substantial anatomical or synaptic plasticity [see also (*63*)]. It is likely that, at a longer time scale, Hebbian plasticity mechanisms could produce more substantial adult reorganization (*64*). Still, deprivation-driven remapping has not been limited to only long time scales. For example, enlargement of receptive fields following deprivation in animal studies has been replicated for amputation (*24*, *65*, *66*) and anesthesia (*15*, *24*, *67*), sometimes after less than 1 hour after intervention. In humans, representational change in response to local blocks has also been reported by several studies [see (*39*) for review] (*68*–*70*). Work by Bjorkman *et al*. (*68*), for example, suggests that anesthetic cream on the forearm leads to expansion of the contralateral hand area. Other human studies suggest that enhanced tactile stimulation of fingers can cause rapid receptive field changes (*42*). However, these changes are also reversible, indicating that these are within the hand area’s representational dynamic range. It is possible that short-term reorganization studies may overestimate reorganization because, while each neuron’s suprathreshold responses are highly plastic, the widespread arborization of afferent connections providing the excitatory input is more stable (*71*). Ultimately, the traditional receptive field is created by inhibitory networks dynamically determining which responses cross the threshold. Hence, it is possible that the full consequences of local unmasking can only be captured in longer time scales. In this context, our present findings highlight the need to apply more caution when interpreting absolute selectivity changes that drive boundary changes within a topographic map.

It is also possible that we did not observe expanded receptive fields in our study, i.e., no increase in activity for neighboring fingers in the D2 cluster, because fMRI pools many cells’ response profiles, so our observed distributed activity, even at baseline, might have already been less than fully inhibited (and thus measurable). We also note that the degree of interfinger convergence likely varies across cortical layers (*72*), which we are unable to tease apart with standard fMRI. Seven-tesla BOLD fMRI is particularly skewed toward processes occurring in the superficial layers (*73*). Regardless, while differences in methodology may drive some discrepancies with the animal literature, previous accounts for plasticity following localized S1 input loss, in the form of spatial remapping, transcends methodologies [e.g., MEG (*37*), and, in squirrel monkeys, optical imaging (*38*), electroencephalography (*74*), and TMS (*75*)]. In contrast, the reduced activity observed across the hand area in our study—seen using a classical univariate approach that is well validated against electrophysiology (Fig. 2B) (*76*)—is another example of this seemingly ubiquitous phenomenon. Ultimately, while methodological differences are substantial, we believe that the organizing principle highlighted here cannot be excused as a mere consequence of using fMRI versus electrophysiology.

In conclusion, the characterization of S1 as a patchwork of isolated clusters representing individual fingers (i.e., a topographic map) has been useful in progressing our understanding of functional hand representation. However, as our results highlight, this overemphasis on selectivity may bias our research into brain plasticity. In particular, complex and behaviorally relevant interactions exist between neighboring representations in the periphery, cortex, and in between; but because these interdependencies between finger representations are normally overshadowed by finger selectivity, they have often been overlooked in previous research on brain remapping and reorganization. When taking these representational motifs into consideration, we find that a drastic and highly localized change to the periphery causes hand-wide and largely homogeneous suppression of cortical hand representation. Our findings are consistent with mounting recent evidence, demonstrating that the representation of an insensate or even an amputated hand is stable and can be evoked by S1 stimulation (*25*). Beyond advancing our understanding of the underlying representational structure of hand representation and (lack of) reorganization, which, in itself, may not generalize to long-term input loss, our study makes two additional contributions that are generalizable. First, from a methodological standpoint, our findings highlight the need to apply more caution when interpreting boundary changes to topographic maps, based on net selectivity. Specifically, because we were able to reproduce this form of remapping while providing strong evidence for representational stability, we suggest that this measure does not necessarily indicate reorganization, i.e., a change away from a norm. Furthermore, from an applied biomedical perspective, if verified as a more global underlying representational feature of S1, the shared representation across fingers (and perhaps even body parts) provides neuroengineers with the ability to complete missing input in certain contexts, for example, following localized brain tissue damage or peripheral injuries. Our findings therefore open up previously unidentified opportunities for restorative applications and brain-computer interface control.

## METHODS

Because the methodology of this study overlaps with that of a previous study (*32*), some subsections (e.g., “Task-based fMRI analysis,” “Localizer and cluster definition,” and “Representational similarity analysis” sections) have been repeated from there.

### Participants

Fifteen healthy volunteers (six female; age = 26.44 ± 1.04) participated in this study. In addition, one volunteer (P16) participated in session 1 but did not complete session 2; their imaging data have not been included, but we did include the collected behavioral data. All participants but one were right-handed, and all experimental tasks were performed using the right hand. All participants gave written informed consent, and ethical approval for the study was obtained from the Health Research Authority, UK (13/SC/0502).

One participant was excluded from the passive multivariate task analysis because they were identified as an outlier (score > 3 SDs below the group mean in the baseline session). This participant was included in the active condition where their scores were considered to be within normal group variance (0.3 SDs above group mean). In addition, because of multiple technical challenges with data acquisition (due to the complex MRI protocols, the induction of the nerve block, and the time-consuming behavioral testing) and the exceedingly long testing session, we sustained some impartial datasets. Fourteen participants took part in the postscan grating orientation task, but two missed one of the two sessions. Thirteen participants had resting-state data for both sessions, and 10 participants had stable magnetic resonance spectra in both sessions (see below for more details). One outlier participant was excluded from the tactile acuity measurements because they were reportedly distracted during the baseline session (Fig. 1, B and C), but group inference was not substantially different when they were included.

### Experimental procedures

Participants attended two sessions that were similarly structured (see Fig. 1A). A finger nerve block was administered in one of the two sessions (in counterbalanced order between participants). In both scan sessions, participants completed an active and a passive task, a resting state, and spectroscopy scans. In the baseline session, participants also performed a functional localizer task that was used to define the finger-selective clusters.

### Procedures outside the scanner

#### Pharmacological nerve block

Seven participants received the nerve block in the first session and eight received it in the second session. The nerve block consisted of a mixture of 2 ml of 2% lidocaine and 2 ml of 0.5% bupivacaine hydrochloride, allowing for both a rapid onset of the anesthetic effect and a stable effect in 5 to 8 hours (depending on blood circulation). The solution was injected by a trained medical professional around the base of the right index finger, and each side of the finger was injected with approximately half of the solution. This thereby formed a ring block, anesthetizing the entire finger.

#### Psychophysical testing

We used a range of tactile acuity and sensitivity assessments to verify the perceptual effectiveness of the nerve block. All measures were carried out during both the baseline and block sessions. To test acuity, an experimenter applied a plastic domed grating with a grating width of 3.5 mm to the glabrous surface of the right distal index finger perpendicularly for a period of approximately 1 s. The grating was oriented vertically or horizontally, randomly over 20 trials, and participants reported the perceived orientation via a mouse click (with their left hand). This provided a relatively quick means to probe tactile acuity and was carried out at four time points: an initial test shortly after the injection, immediately before the scan, immediately after the scan, and after the more precise postscan sensitivity tests (see Fig. 1A). Performance above 70% correct in the prescanning test of the block session was an a priori criterion for exclusion. On the basis of this criterion, all participants were successfully blocked. We also tested the adjacent D3 to confirm that the effects of the nerve block were localized.

After the injection (but before the scan), we also assessed tactile sensitivity using Von Frey hairs. Two hairs of different forces (2.0 and 8.0 mN) were applied to the glabrous surface of the distal index finger. Participants were asked to indicate which force felt stronger in each trial, over 20 trials. On each trial, the filament was pressed perpendicularly against the fingertip for approximately 2 s.

After the scan, we estimated tactile acuity thresholds more precisely for the index, middle, and ring fingers. Plastic domed gratings with five different grating widths (0.5, 1.0, 1.5, 2.5, and 3.5 mm) were briefly applied to each of the finger’s distal pad in either horizontal or vertical orientation, over 20 trials per grating width and finger. Participants were asked to indicate the orientation of the grating with a mouse click of the left hand. Participants were blindfolded throughout the procedure. The order in which the gratings were presented was randomized. The trials were grouped in two blocks interspersed by a short break. To encourage engagement, participants received intermittent auditory feedback on their performance (percentage correct) over headphones.

### MRI tasks

#### General procedures

In a single session, participants completed an active and a passive task in the scanner (in counterbalanced order between participants), as well as an anatomical scan, a resting state scan, and an MRS scan (also performed during rest). The active and passive tasks were completed over four separate runs (for each task), as described in (*32*).

The scan protocol was identical for the passive and active task. Each run is composed of individual finger blocks for each of the five fingers (one tap per second, 12-s blocks) of the right hand, as well as no movement (rest) blocks lasting 12 or 24 s. Each condition was repeated three times in a semicounterbalanced order within each run, here termed “random design.” Each run totaled 118 volumes and comprised a different block order.

We also conducted a functional localizer before the active task in the baseline session to independently identify finger-selective regions of interest (here termed clusters C1 to C5). Two runs were acquired, with a reversed order from each other, each consisting of 108 volumes, covering 5 cycles around the hand. Last, 150 resting-state volumes were collected in both sessions. For more details, see Supplementary Methods.

While prolonged stimulation may cause habituation, our study involved identical stimulation across conditions and order was counterbalanced. Thus, while habituation could cause activity to decrease or, theoretically, could cause a drop in finger dissimilarity, we do not believe that habituation could have caused the differences in baseline and block sessions that we show here.

### Tactile perceptual analysis

For the sensitivity checks of grating orientation judgment (Fig. 1, B and C), the four measurements were grouped into prescan (two tests) and postscan (two tests) measurements. Session comparison was done on the average percentage of correct responses. For the detection performance with the Von Frey hairs (Fig. 1D), raw accuracy data were *z*-normalized and *d* prime (*d*′) scores were calculated (hits minus false alarms).

Tactile psychophysical thresholds were determined separately for each finger/session, using standard procedures [e.g., (*39*)]. In short, this was done by plotting accuracy as a function of grating size across all levels of stimulus difficulty and fitting a Weibull curve using a least-squares function in MATLAB (two free parameters: gamma = 0.05, lambda = 0). The threshold for this psychometric function was interpolated from the grating size estimated to yield 82% accuracy. We also extracted the slope and goodness of fit values (*R*^2^) for each curve fit (the slope is taken as the steepness of the psychometric function at the threshold point, and the *R*^2^ represents how well the psychometric function represented the data, i.e., how close the data points are to the line). Overall, the psychometric functions predicted the data with relative accuracy (average *R*^2^ = 0.76, SEM = 0.02). Thresholds were compared across fingers and sessions.

### MRI acquisition and preprocessing

For details on the acquisition and preprocessing of the MRI images, see Supplementary Methods.

### Task-based fMRI analysis

#### Active and passive tasks

A voxel-based GLM analysis was carried out on each of the 16 runs (4 active and 4 passive per session) using FEAT to identify activity patterns for each finger condition. The design was convolved with the double-gamma hemodynamic response function, as well as its temporal derivative. For each run, 11 contrasts were set up: each finger versus rest, each finger versus all other fingers, and all fingers versus rest. For univariate analysis, the estimates from the four active/passive runs were then averaged voxel wise for each participant using a fixed effects model, creating 11 main activity patterns for each task. These contrasts were used for the follow-up analysis described below.

#### Localizer and cluster definition

The traveling wave task described above was used to identify finger-specific voxel clusters. The analysis was carried out as in (*28*). In short, a reference model was created using a convolved hemodynamic response function to account for the hemodynamic response. This model consisted of an 8-s “on” period followed by a 32-s “off” period to model the movement block of one finger for 1 cycle. The model was shifted by 2 seconds (i.e., the acquisition time of one volume) 20 times to model a single-movement cycle (which lasted 40 s), thus resulting in 20 reference models. This was repeated five times to model the 5 cycles in each run. Each of these reference models was then correlated with each voxel’s preprocessed BOLD signal time course. This resulted in cross-correlation *r* values for each voxel, which were standardized using the Fisher’s *r*-to-*z* transformation. Lags were assigned to each finger (four lags per finger) to average the *r* values across runs for each voxel. This resulted in an *r* value for each finger that was further averaged across the forward and backward runs. To define finger specificity, each voxel was assigned to one finger using a winner-takes-all approach. This was done by finding the maximum for each voxel across the five averaged *r* values and assigning the voxel to the corresponding finger.

To correct for multiple comparisons, a false discovery rate (FDR) threshold (*q* < 0.01) was applied to each finger individually [as in (*28*)]. The resulting FDR-corrected finger-specific voxels were then used to create finger-specific clusters showing strong finger selectivity for each of the fingers. This was done by using an anatomically defined mask of the S1 hand area that was defined for each participant on the basis of a FreeSurfer structural segmentation of S1 subdivisions. Brodmann areas 3a, 3b, and 1, spanning a 2-cm strip dorsal/ventral to the anatomical hand knob, were included in the mask (*29*). The finger-specific activity under this mask was used to create finger-specific S1 clusters (C1 to C5; see, e.g., Fig. 2). This approach allowed us to identify finger-specific S1 clusters on an individual participant basis, which were used for further analysis of the active and passive tasks. Clusters are not necessarily contiguous. The size of these clusters varied considerably between participants, as reported in table S1. Although the FreeSurfer structural segmentation cannot indisputably separate the subdivisions of S1, we confirmed that the finger-specific clusters lay primarily in areas 3b and 1. Using a lower bound of 95% confidence for the area assignment, 20 to 45% and 30 to 45% of the clusters’ voxels were assigned to areas 3b and 1, respectively (see fig. S4). Considering the difference in anatomical size between the subdivisions (average size area 3b: 1444 voxels; area 1: 2097 voxels), this distribution matches the slight dominance of area 3b in the previous literature. Last, the entire S1 hand mask was also used as its own region of interest for the winner-takes-all (remapping) analysis reported in Fig. 2 (B and C) and the multivariate analysis reported in Fig. 3 (B and C) (excluding highly selective voxels in the clusters).

#### Univariate selectivity analyses and winner-takes-all maps for the main tasks

To examine the distribution and selectivity of finger activity in the active/passive tasks, average activity was calculated for each finger in each finger cluster. Activity was calculated separately for each finger, condition, and session. As a measure of selectivity, we subtracted in each cluster the average activity of the nontarget fingers from that of the target finger (i.e., the finger that the cluster is selective for). For this analysis, as reported in the “Results” section, activity of D2 and activity in cluster C2 were ignored, so each cluster had three nontarget fingers.

To create winner-takes-all maps for the passive task, we first projected the five finger-versus-rest contrasts (averaged across runs) from the passive task onto the cortical surface. Within the S1 hand area (see above), each voxel was either assigned to the finger that evoked the strongest BOLD response or assigned as nonactive if the maximum response did not exceed 0. To simulate classical analysis carried out following amputation, this analysis was repeated while ignoring D2, i.e., voxels could only be assigned to one of four fingers. To examine whether rapid invasion occurred in the deprived cortical territory, we compared the number of voxels assigned to each finger within D2-selective cluster C2.

#### Representational similarity analysis

We used RSA (*77*) to assess the multivariate relationships between the activity patterns generated across fingers and tasks. The (dis)similarity between activity patterns within the S1 hand mask was measured for each finger pair using the cross-validated Mahalanobis distance (*78*). The activity patterns were prewhitened using the residuals from the GLM and then cross-nobis distances were calculated for each task (active/passive) separately, using each pair of imaging runs and averaging those results. Because of cross-validations, the distance value is expected to be zero (but can go below) if two patterns are not statistically different from each other. Otherwise, greater distances indicate larger differences in multivariate representation. The analysis above produced 10 distance values (one for each finger pair) per task/region of interest, forming an RDM per participant. These values can statistically be compared to 0 (i.e., representing no difference between conditions or rest; see the “Statistics” section for more details). This analysis was repeated for three regions of interest: within each finger-selective cluster (Fig. 3A), across the five finger-selective clusters (Fig. 3B), and across the entire hand area, after excluding the finger-selective clusters (Fig. 3C).

As an aid to visualize the RDMs (and not used in any statistical analysis), we also performed MDS. This analysis projects the higher-dimensional RDM into a lower-dimensional space while preserving the interfinger distances as accurately as possible. Rest was included as an extra condition (with uniform activity of 0), so finger-specific differences could be separated from differences from rest. MDS was performed on individual data and averaged across participants after Procrustes alignment (without scaling) to remove any arbitrary rotations introduced by MDS. The two dimensions along which the data were projected showed the maximal between-finger variance, reflecting differences between fingers rather than nonfinger-specific variation from rest.

### Magnetic resonance spectroscopy

For details on MRS, see Supplementary Methods.

### Resting-state scan

Following the same preprocessing pipeline as for the task fMRI (see Supplementary Methods), the mean time course of the resting-state fMRI scan was extracted from the S1 region of interest. The SD of the time series over time was compared between sessions. Less signal variation would suggest diminished excitability that is not task specific.

### Modeling details

Peripheral inputs were generated using TouchSim (*7*), a model that reconstructs typical afferent brain activation to stimuli placed across the hand. For the baseline condition, modeled tapping on each of the five fingertips generated input mimicking that observed in the passive fMRI task. The nerve block was simulated in the model by reducing the activity of all afferents in D2, for all stimulus profiles, to 20% of baseline inputs. Thus, we assume some small residual activation. Peripheral input to a cortical cluster was pooled for each finger.

The cortical model consists of a feedforward part, where each cortical cluster receives input from all fingers. It further involves a set of lateral connections, where each cortical cluster excites or inhibits other clusters. The strength of the lateral excitation and inhibition is determined by the activity of the cortical cluster. Thus, at each time step *t*, the activity of a single cortical cluster can be calculated as

$$c_{i}(t)=\alpha_{i}{w_{i}}^{T}p+b+{l_{i}}^{T}c(t-1)$$

where *c_i_*(*t*) is the activity of cortical cluster *i* responses at time step *t*, α is a gain factor, **w** is a weight vector specifying each finger’s connection strength to the cortical cluster, **p** is the vector of peripheral inputs from each finger, *b* is a scalar offset, **l** is the lateral connection field, and **c**(*t* − 1) is the vector of cortical activity from the previous time step. To simulate cortical activity under peripheral stimulation, we iterated the above equation until the cortical cluster activity had settled, usually within six time steps.

For the baseline model, all gain factors α were set to 1. The lateral connection **l***_ij_* between clusters *i* and *j* depended on the distance between the cortical clusters alone, and, therefore, each cortical cluster had the same lateral connection pattern, only shifted spatially. In the model presented here, we assumed short-range excitation and long-range inhibition. Such a lateral connection pattern is frequently assumed in cortical modeling and can indeed recreate the canonical RSA hand representation commonly observed in S1. However, we note that shifting the “rest state” against which cortical activity is expressed will also change the lateral connectivity pattern and, for example, lead to inhibition between neighboring clusters. We cannot disambiguate between models that only differ by what rest state is assumed, but all these models require both a global and a D2-specific gain shift to mimic the empirical results under block, so our main results are unaffected. In Fig. 4 (C to E), we increased the baseline to allow for the typical pattern of lateral connectivity shown in Fig. 4B. The model parameters **w***_i_* and *b* were fitted to the empirical data in the baseline session, using the TouchSim-generated finger responses. Feedforward weights between the fingers and cortical units were initially fit using multiple regression. Then, an iterative process was used to adjust the weights on the basis of the settled activity after multiple lateral updates. RDMs were calculated using the Euclidean distances between the responses of the modeled cortical units for each finger stimulation.

Using the feedforward weights and lateral connectivity pattern generated using baseline session input, we then calculated the result of D2 block without any additional cortical changes (i.e., the static model). As this led to a poor fit, we let the gain parameters α vary to test whether homeostatic mechanisms might account for the observed changes. We found that two changes were required to reproduce the experimental findings: first, a global reduction in the activity of all fingers (α = 0.75) and, second, an increase in the D2 cortical cluster gain (α_2_ = 1.25). This increase enables widespread changes in the activity of all clusters via propagation through the lateral connections and stabilizes the cortical representation of D2.

#### Statistics

Unless stated otherwise, statistical comparisons were done using (paired or one-sample) two-tailed *t* tests and (repeated-measures) analysis of variance (ANOVA). Bulk *t* tests on individual finger clusters were not corrected for multiple comparisons to minimize false negatives because of a lack of power. This decision may have increased the false-positive error of our results. To confirm the null hypothesis for key nonsignificant results, Bayesian statistics was carried out, as implemented in JASP (version 0.13.1; using as a prior a Cauchy distribution centered on 0 with a width of 0.707). A Bayes factor (for the alternative over null hypothesis; BF10) of less than 0.33 was considered as substantial evidence in favor of the null (*79*).

The significance of rank correlations between the canonical representational finger pattern and nontarget fingers in each cluster was calculated by comparing the sample mean against that of permuted labels. These tests were not corrected for multiple comparisons because inference was done on a higher-level result, i.e., the number of clusters with a canonical finger order. Last, to estimate tactile acuity and sensitivity thresholds, we used GEEs (generalized estimating equations).

## Supplementary Materials

### This PDF file includes:

Supplementary Results Figs. S1 to S4 Tables S1 and S2 Supplementary Methods References

## Notes

View/request a protocol for this paper from *Bio-protocol*.

## References

[1] J. H. Kaas, R. J. Nelson, M. Sur, C.-S. Lin, M. M. Merzenich, Multiple representations of the body within the primary somatosensory cortex of primates. Science 204, 521–523 (1979). 107591 10.1126/science.107591

[2] R. M. Sanchez-Panchuelo, S. Francis, R. Bowtell, D. Schluppeck, Mapping human somatosensory cortex in individual subjects with 7T functional MRI. J. Neurophysiol. 103, 2544–2556 (2010). 20164393 10.1152/jn.01017.2009 PMC2867563

[3] P. H. Thakur, P. J. Fitzgerald, S. S. Hsiao, Second-order receptive fields reveal multidigit interactions in area 3b of the macaque monkey. J. Neurophysiol. 108, 243–262 (2012). 22457468 10.1152/jn.01022.2010 PMC3434610

[4] T. M. McKenna, B. L. Whitsel, D. A. Dreyer, Anterior parietal cortical topographic organization in macaque monkey: A reevaluation. J. Neurophysiol. 48, 289–317 (1982). 7119852 10.1152/jn.1982.48.2.289

[5] M. Tommerdahl, O. V. Favorov, B. L. Whitsel, Dynamic representations of the somatosensory cortex. Neurosci. Biobehav. Rev. 34, 160–170 (2010). 19732790 10.1016/j.neubiorev.2009.08.009

[6] L. R. Manfredi, A. T. Baker, D. O. Elias, J. F. Dammann, M. C. Zielinski, V. S. Polashock, S. J. Bensmaia, The effect of surface wave propagation on neural responses to vibration in primate glabrous skin. PLOS ONE 7, e31203 (2012). 22348055 10.1371/journal.pone.0031203 PMC3278420

[7] H. P. Saal, B. P. Delhaye, B. C. Rayhaun, S. J. Bensmaia, Simulating tactile signals from the whole hand with millisecond precision. Proc. Natl. Acad. Sci. U.S.A. 114, E5693–E5702 (2017). 28652360 10.1073/pnas.1704856114 PMC5514748

[8] Y. Shao, V. Hayward, Y. Visell, Compression of dynamic tactile information in the human hand. Sci. Adv. 6, eaaz1158 (2020). 32494610 10.1126/sciadv.aaz1158 PMC7159916

[9] N. Ejaz, M. Hamada, J. Diedrichsen, Hand use predicts the structure of representations in sensorimotor cortex. Nat. Neurosci. 18, 1034–1040 (2015). 26030847 10.1038/nn.4038

[10] H. Dempsey-Jones, V. Harrar, J. Oliver, H. Johansen-Berg, C. Spence, T. R. Makin, Transfer of tactile perceptual learning to untrained neighbouring fingers reflects natural use relationships. J. Neurophysiol. 115, 1088–1097 (2016). 26631145 10.1152/jn.00181.2015 PMC4808091

[11] E. Kuehn, B. Pleger, Encoding schemes in somatosensation: From micro- to meta-topography. Neuroimage 223, 117255 (2020). 32800990 10.1016/j.neuroimage.2020.117255

[12] D. Muret, V. Root, P. Kieliba, D. Clode, T. R. Makin, Beyond body maps: Information content of specific body parts is distributed across the somatosensory homunculus. Cell Rep. 38, 110523 (2022). 35294887 10.1016/j.celrep.2022.110523 PMC8938902

[13] D. V. Buonomano, M. M. Merzenich, Cortical plasticity: From synapses to maps. Annu. Rev. Neurosci. 21, 149–186 (1998). 9530495 10.1146/annurev.neuro.21.1.149

[14] M. M. Merzenich, J. H. Kaas, J. T. Wall, M. Sur, R. J. Nelson, D. J. Felleman, Progression of change following median nerve section in the cortical representation of the hand in areas 3b and 1 in adult owl and squirrel monkeys. Neuroscience 10, 639–665 (1983). 6646426 10.1016/0306-4522(83)90208-7

[15] B. M. Faggin, K. T. Nguyen, M. A. Nicolelis, Immediate and simultaneous sensory reorganization at cortical and subcortical levels of the somatosensory system. Proc. Natl. Acad. Sci. U.S.A. 94, 9428–9433 (1997). 9256499 10.1073/pnas.94.17.9428 PMC23207

[16] T. P. Pons, P. E. Garraghty, A. K. Ommaya, J. H. Kaas, E. Taub, M. Mishkin, Massive cortical reorganization after sensory deafferentation in adult macaques. Science 252, 1857–1860 (1991). 1843843 10.1126/science.1843843

[17] J. D. Churchill, N. Muja, W. A. Myers, J. Besheer, P. E. Garraghty, Somatotopic consolidation: A third phase of reorganization after peripheral nerve injury in adult squirrel monkeys. Exp. Brain Res. 118, 189–196 (1998). 9547087 10.1007/s002210050271

[18] M. M. Merzenich, R. J. Nelson, M. P. Stryker, M. S. Cynader, A. Schoppmann, J. M. Zook, Somatosensory cortical map changes following digit amputation in adult monkeys. J Comp Neurol 224, 591–605 (1984). 6725633 10.1002/cne.902240408

[19] M. M. Merzenich, J. H. Kaas, J. Wall, R. J. Nelson, M. Sur, D. Felleman, Topographic reorganization of somatosensory cortical areas 3b and 1 in adult monkeys following restricted deafferentation. Neuroscience 8, 33–55 (1983). 6835522 10.1016/0306-4522(83)90024-6

[20] D. D. Rasmusson, Reorganization of raccoon somatosensory cortex following removal of the fifth digit. J. Comp. Neurol. 205, 313–326 (1982). 7096623 10.1002/cne.902050402

[21] J. H. Kaas, Plasticity of sensory and motor maps in adult mammals. Annu. Rev. Neurosci. 14, 137–167 (1991). 2031570 10.1146/annurev.ne.14.030191.001033

[22] N. M. Weinberger, Dynamic regulation of receptive fields and maps in the adult sensory cortex. Annu. Rev. Neurosci. 18, 129–158 (1995). 7605058 10.1146/annurev.ne.18.030195.001021 PMC3621971

[23] D. E. Feldman, M. Brecht, Map plasticity in somatosensory cortex. Science 310, 810–815 (2005). 16272113 10.1126/science.1115807

[24] M. B. Calford, R. Tweedale, Immediate expansion of receptive fields of neurons in area 3b of macaque monkeys after digit denervation. Somatosens. Mot. Res. 8, 249–260 (1991). 1767621 10.3109/08990229109144748

[25] T. R. Makin, S. J. Bensmaia, Stability of sensory topographies in adult cortex. Trends Cogn. Sci. 21, 195–204 (2017). 28214130 10.1016/j.tics.2017.01.002 PMC6052795

[26] S. N. Flesher, J. E. Downey, J. M. Weiss, C. L. Hughes, A. J. Herrera, E. C. Tyler-Kabara, M. L. Boninger, J. L. Collinger, R. A. Gaunt, A brain-computer interface that evokes tactile sensations improves robotic arm control. Science 372, 831–836 (2021). 34016775 10.1126/science.abd0380 PMC8715714

[27] S. Kikkert, D. Pfyffer, M. Verling, P. Freund, N. Wenderoth, Finger somatotopy is preserved after tetraplegia but deteriorates over time. eLife 10, e67713 (2021). 34665133 10.7554/eLife.67713 PMC8575460

[28] S. Kikkert, J. Kolasinski, S. Jbabdi, I. Tracey, C. F. Beckmann, H. J. Berg, T. R. Makin, Revealing the neural fingerprints of a missing hand. Elife 5, e15292 (2016). 27552053 10.7554/eLife.15292 PMC5040556

[29] D. B. Wesselink, F. M. Z. van den Heiligenberg, N. Ejaz, H. Dempsey-Jones, L. Cardinali, A. Tarall-Jozwiak, J. Diedrichsen, T. R. Makin, Obtaining and maintaining cortical hand representation as evidenced from acquired and congenital handlessness. eLife 8, e37227 (2019). 30717824 10.7554/eLife.37227 PMC6363469

[30] M. Bruurmijn, I. P. L. Pereboom, M. J. Vansteensel, M. A. H. Raemaekers, N. F. Ramsey, Preservation of hand movement representation in the sensorimotor areas of amputees. Brain 140, 3166–3178 (2017). 29088322 10.1093/brain/awx274 PMC6411136

[31] A. Serino, M. Akselrod, R. Salomon, R. Martuzzi, M. L. Blefari, E. Canzoneri, G. Rognini, W. van der Zwaag, M. Iakova, F. Luthi, A. Amoresano, T. Kuiken, O. Blanke, Upper limb cortical maps in amputees with targeted muscle and sensory reinnervation. Brain 140, 2993–3011 (2017). 29088353 10.1093/brain/awx242

[32] Z.-B. Sanders, D. B. Wesselink, H. Dempsey-Jones, T. R. Makin, Similar somatotopy for active and passive digit representation in primary somatosensory cortex. bioRxiv 754648 [Preprint]. 5 September 2019. 10.1101/754648. PMC10203813 37145934

[33] S. Raymond, A. Gissen, Mechanisms of Differential Nerve Block, in *Local Anesthetics*, G. R. Strichartz, Ed. (Springer, 1987), pp. 95–164.

[34] C. Mehring, M. Akselrod, L. Bashford, M. Mace, H. Choi, M. Blüher, A.-S. Buschhoff, T. Pistohl, R. Salomon, A. Cheah, O. Blanke, A. Serino, E. Burdet, Augmented manipulation ability in humans with six-fingered hands. Nat. Commun. 10, 2401 (2019). 31160580 10.1038/s41467-019-10306-w PMC6547737

[35] P. Kieliba, D. Clode, R. O. Maimon-Mor, T. R. Makin, Robotic hand augmentation drives changes in neural body representation. Sci. Robot. 6, eabd7935 (2021). 34043536 10.1126/scirobotics.abd7935 PMC7612043

[36] C. J. Stagg, S. Bestmann, A. O. Constantinescu, L. M. Moreno, C. Allman, R. Mekle, M. Woolrich, J. Near, H. Johansen-Berg, J. C. Rothwell, Relationship between physiological measures of excitability and levels of glutamate and GABA in the human motor cortex. J. Physiol. 589, 5845–5855 (2011). 22005678 10.1113/jphysiol.2011.216978 PMC3249054

[37] H. Flor, T. Elbert, S. Knecht, C. Wienbruch, C. Pantev, N. Birbaumers, W. Larbig, E. Taub, Phantom-limb pain as a perceptual correlate of cortical reorganization following arm amputation. Nature 375, 482–484 (1995). 7777055 10.1038/375482a0

[38] L. M. Chen, H. X. Qi, J. H. Kaas, Dynamic reorganization of digit representations in somatosensory cortex of nonhuman primates after spinal cord injury. J. Neurosci. 32, 14649–14663 (2012). 23077051 10.1523/JNEUROSCI.1841-12.2012 PMC3498942

[39] H. Dempsey-Jones, A. C. Themistocleous, D. Carone, T. W. C. Ng, V. Harrar, T. R. Makin, Blocking tactile input to one finger using anaesthetic enhances touch perception and learning in other fingers. J. Exp. Psychol. 148, 713–727 (2019). 10.1037/xge0000514 PMC6459089 30973263

[40] J. Xing, G. L. Gerstein, Simulation of dynamic receptive fields in primary visual cortex. Vision Res. 34, 1901–1911 (1994). 7941392 10.1016/0042-6989(94)90314-x

[41] K. Ogawa, K. Mitsui, F. Imai, S. Nishida, Long-term training-dependent representation of individual finger movements in the primary motor cortex. Neuroimage 202, 116051 (2019). 31351164 10.1016/j.neuroimage.2019.116051

[42] B. Godde, F. Spengler, H. R. Dinse, Associative pairing of tactile stimulation induces somatosensory cortical reorganization in rats and humans. Neuroreport 8, 281–285 (1996). 9051796 10.1097/00001756-199612200-00056

[43] N. Kambi, P. Halder, R. Rajan, V. Arora, P. Chand, M. Arora, N. Jain, Large-scale reorganization of the somatosensory cortex following spinal cord injuries is due to brainstem plasticity. Nat. Commun. 5, 3602 (2014). 24710038 10.1038/ncomms4602

[44] J. A. Pruszynski, R. S. Johansson, Edge-orientation processing in first-order tactile neurons. Nat. Neurosci. 17, 1404–1409 (2014). 25174006 10.1038/nn.3804

[45] H. P. Saal, M. A. Harvey, S. J. Bensmaia, Rate and timing of cortical responses driven by separate sensory channels. eLife 4, e10450 (2015). 26650354 10.7554/eLife.10450 PMC4755746

[46] J. N. Ingram, K. P. Kording, I. S. Howard, D. M. Wolpert, The statistics of natural hand movements. Exp. Brain Res. 188, 223–236 (2008). 18369608 10.1007/s00221-008-1355-3 PMC2636901

[47] R. M. Sanchez Panchuelo, R. Ackerley, P. M. Glover, R. W. Bowtell, J. Wessberg, S. T. Francis, F. McGlone, Mapping quantal touch using 7 Tesla functional magnetic resonance imaging and single-unit intraneural microstimulation. eLife 5, e12812 (2016). 27154626 10.7554/eLife.12812 PMC4898929

[48] G. G. Turrigiano, S. B. Nelson, Hebb and homeostasis in neuronal plasticity. Curr. Opin. Neurobiol. 10, 358–364 (2000). 10851171 10.1016/s0959-4388(00)00091-x

[49] F. Zenke, W. Gerstner, S. Ganguli, The temporal paradox of Hebbian learning and homeostatic plasticity. Curr. Opin. Neurobiol. 43, 166–176 (2017). 28431369 10.1016/j.conb.2017.03.015

[50] E. Castaldi, C. Lunghi, M. C. Morrone, Neuroplasticity in adult human visual cortex. Neurosci. Biobehav. Rev. 112, 542–552 (2020). 32092315 10.1016/j.neubiorev.2020.02.028

[51] B. Nystrom, K. E. Hagbarth, Microelectrode recordings from transected nerves in amputees with phantom limb pain. Neurosci. Lett. 27, 211–216 (1981). 7322453 10.1016/0304-3940(81)90270-6

[52] S. Lee, G. E. Carvell, D. J. Simons, Motor modulation of afferent somatosensory circuits. Nat. Neurosci. 11, 1430–1438 (2008). 19011625 10.1038/nn.2227 PMC2597103

[53] R. A. Adams, S. Shipp, K. J. Friston, Predictions not commands: Active inference in the motor system. Brain Struct. Funct. 218, 611–643 (2013). 23129312 10.1007/s00429-012-0475-5 PMC3637647

[54] K. D. Davis, Z. H. Kiss, L. Luo, R. R. Tasker, A. M. Lozano, J. O. Dostrovsky, Phantom sensations generated by thalamic microstimulation. Nature 391, 385–387 (1998). 9450753 10.1038/34905

[55] E. Kuehn, P. Haggard, A. Villringer, B. Pleger, M. I. Sereno, Visually-driven maps in area 3b. J. Neurosci. 38, 1295–1310 (2018). 29301873 10.1523/JNEUROSCI.0491-17.2017 PMC6596270

[56] M. Jafari, T. Aflalo, S. Chivukula, S. S. Kellis, M. A. Salas, S. L. Norman, K. Pejsa, C. Y. Liu, R. A. Andersen, The human primary somatosensory cortex encodes imagined movement in the absence of sensory information. Commun. Biol. 3, 757 (2020). 33311578 10.1038/s42003-020-01484-1 PMC7732821

[57] A. M. Puckett, S. Bollmann, M. Barth, R. Cunnington, Measuring the effects of attention to individual fingertips in somatosensory cortex using ultra-high field (7T) fMRI. Neuroimage 161, 179–187 (2017). 28801252 10.1016/j.neuroimage.2017.08.014

[58] F. W. Smith, L. Muckli, Nonstimulated early visual areas carry information about surrounding context. Proc. Natl. Acad. Sci. U.S.A. 107, 20099–20103 (2010). 21041652 10.1073/pnas.1000233107 PMC2993348

[59] F. P. de Lange, M. Heilbron, P. Kok, How do expectations shape perception? Trends Cogn. Sci. 22, 764–779 (2018). 30122170 10.1016/j.tics.2018.06.002

[60] J. L. Reed, P. Pouget, H.-X. Qi, Z. Zhou, M. R. Bernard, M. J. Burish, J. Haitas, A. B. Bonds, J. H. Kaas, Widespread spatial integration in primary somatosensory cortex. Proc. Natl. Acad. Sci. U.S.A. 105, 10233–10237 (2008). 18632579 10.1073/pnas.0803800105 PMC2481381

[61] D. Muret, T. R. Makin, The homeostatic homunculus: Rethinking deprivation-triggered reorganisation. Curr. Opin. Neurobiol. 67, 115–122 (2021). 33248404 10.1016/j.conb.2020.08.008

[62] T. R. Makin, H. Flor, Brain (re)organisation following amputation: Implications for phantom limb pain. Neuroimage 218, 116943 (2020). 32428706 10.1016/j.neuroimage.2020.116943 PMC7422832

[63] D. D. Rasmusson, D. M. Nance, Non-overlapping thalamocortical projections for separate forepaw digits before and after cortical reorganization in the raccoon. Brain Res. Bull. 16, 399–406 (1986). 3011221 10.1016/0361-9230(86)90063-8

[64] D. E. Feldman, Synaptic mechanisms for plasticity in neocortex. Annu. Rev. Neurosci. 32, 33–55 (2009). 19400721 10.1146/annurev.neuro.051508.135516 PMC3071739

[65] B. G. Turnbull, D. D. Rasmusson, Acute effects of total or partial digit denervation on raccoon somatosensory cortex. Somatosens. Mot. Res. 7, 365–389 (1990). 1963250 10.3109/08990229009144714

[66] R. C. Kolarik, S. K. Rasey, J. T. Wall, The consistency, extent, and locations of early-onset changes in cortical nerve dominance aggregates following injury of nerves to primate hands. J. Neurosci. 14, 4269–4288 (1994). 8027778 10.1523/JNEUROSCI.14-07-04269.1994 PMC6577019

[67] F. Panetsos, A. Nunez, C. Avendano, Local anaesthesia induces immediate receptive field changes in nucleus gracilis and cortex. Neuroreport 7, 150–152 (1995). 8742439

[68] A. Bjorkman, A. Weibull, B. Rosen, J. Svensson, G. Lundborg, Rapid cortical reorganisation and improved sensitivity of the hand following cutaneous anaesthesia of the forearm. Eur. J. Neurosci. 29, 837–844 (2009). 19250441 10.1111/j.1460-9568.2009.06629.x

[69] T. Weiss, W. H. Miltner, J. Liepert, W. Meissner, E. Taub, Rapid functional plasticity in the primary somatomotor cortex and perceptual changes after nerve block. Eur. J. Neurosci. 20, 3413–3423 (2004). 15610174 10.1111/j.1460-9568.2004.03790.x

[70] K. J. Werhahn, J. Mortensen, R. W. Van Boven, K. E. Zeuner, L. G. Cohen, Enhanced tactile spatial acuity and cortical processing during acute hand deafferentation. Nat. Neurosci. 5, 936–938 (2002). 12219095 10.1038/nn917

[71] M. B. Calford, Dynamic representational plasticity in sensory cortex. Neuroscience 111, 709–738 (2002). 12031401 10.1016/s0306-4522(02)00022-2

[72] M. Sur, P. E. Garraghty, C. J. Bruce, Somatosensory cortex in macaque monkeys: Laminar differences in receptive field size in areas 3b and 1. Brain Res. 342, 391–395 (1985). 4041845 10.1016/0006-8993(85)91144-8

[73] J. Goense, H. Merkle, N. K. Logothetis, High-resolution fMRI reveals laminar differences in neurovascular coupling between positive and negative BOLD responses. Neuron 76, 629–639 (2012). 23141073 10.1016/j.neuron.2012.09.019 PMC5234326

[74] A. Karl, N. Birbaumer, W. Lutzenberger, L. G. Cohen, H. Flor, Reorganization of motor and somatosensory cortex in upper extremity amputees with phantom limb pain. J. Neurosci. 21, 3609–3618 (2001). 11331390 10.1523/JNEUROSCI.21-10-03609.2001 PMC6762494

[75] A. Pascual-Leone, M. Peris, J. M. Tormos, A. P. Pascual, M. D. Catalá, Reorganization of human cortical motor output maps following traumatic forearm amputation. Neuroreport 7, 2068–2070 (1996). 8930960 10.1097/00001756-199609020-00002

[76] R. Mukamel, H. Gelbard, A. Arieli, U. Hasson, I. Fried, R. Malach, Coupling between neuronal firing, field potentials, and FMRI in human auditory cortex. Science 309, 951–954 (2005). 16081741 10.1126/science.1110913

[77] N. Kriegeskorte, M. Mur, P. Bandettini, Representational similarity analysis—Connecting the branches of systems neuroscience. Front. Syst. Neurosci. 2, 4 (2008). 19104670 10.3389/neuro.06.004.2008 PMC2605405

[78] A. Walther, H. Nili, N. Ejaz, A. Alink, N. Kriegeskorte, J. Diedrichsen, Reliability of dissimilarity measures for multi-voxel pattern analysis. Neuroimage 137, 188–200 (2016). 26707889 10.1016/j.neuroimage.2015.12.012

[79] R. Wetzels, D. Matzke, M. D. Lee, J. N. Rouder, G. J. Iverson, E. J. Wagenmakers, Statistical evidence in experimental psychology: An empirical comparison using 855 t tests. Perspect. Psychol. Sci. 6, 291–298 (2011). 26168519 10.1177/1745691611406923

[80] S. Moeller, E. Yacoub, C. A. Olman, E. Auerbach, J. Strupp, N. Harel, K. Uğurbil, Multiband multislice GE-EPI at 7 tesla, with 16-fold acceleration using partial parallel imaging with application to high spatial and temporal whole-brain fMRI. Magn. Reson. Med. 63, 1144–1153 (2010). 20432285 10.1002/mrm.22361 PMC2906244

[81] K. Ugurbil, J. Xu, E. J. Auerbach, S. Moeller, A. T. Vu, J. M. Duarte-Carvajalino, C. Lenglet, X. Wu, S. Schmitter, P. F. Van de Moortele, J. Strupp, G. Sapiro, F. De Martino, D. Wang, N. Harel, M. Garwood, L. Chen, D. A. Feinberg, S. M. Smith, K. L. Miller, S. N. Sotiropoulos, S. Jbabdi, J. L. R. Andersson, T. E. J. Behrens, M. F. Glasser, D. C. Van Essen, E. Yacoub; WU-Minn HCP Consortium, Pushing spatial and temporal resolution for functional and diffusion MRI in the Human Connectome Project. Neuroimage 80, 80–104 (2013). 23702417 10.1016/j.neuroimage.2013.05.012 PMC3740184

[82] C. Lunghi, U. E. Emir, M. C. Morrone, H. Bridge, Short-term monocular deprivation alters GABA in the adult human visual cortex. Curr. Biol. 25, 1496–1501 (2015). 26004760 10.1016/j.cub.2015.04.021 PMC5040500

[83] A. M. Dale, B. Fischl, M. I. Sereno, Cortical surface-based analysis. I. Segmentation and surface reconstruction. Neuroimage 9, 179–194 (1999). 9931268 10.1006/nimg.1998.0395

[84] M. Jenkinson, C. F. Beckmann, T. E. J. Behrens, M. W. Woolrich, S. M. Smith, FSL. Neuroimage 62, 782–790 (2012). 21979382 10.1016/j.neuroimage.2011.09.015

[85] M. Jenkinson, P. Bannister, M. Brady, S. Smith, Improved optimization for the robust and accurate linear registration and motion correction of brain images. Neuroimage 17, 825–841 (2002). 12377157 10.1016/s1053-8119(02)91132-8

[86] S. M. Smith, Fast robust automated brain extraction. Hum. Brain Mapp. 17, 143–155 (2002). 12391568 10.1002/hbm.10062 PMC6871816

[87] M. Jenkinson, S. Smith, A global optimisation method for robust affine registration of brain images. Med. Image Anal. 5, 143–156 (2001). 11516708 10.1016/s1361-8415(01)00036-6

[88] R. Martuzzi, W. van der Zwaag, J. Farthouat, R. Gruetter, O. Blanke, Human finger somatotopy in areas 3b, 1, and 2: A 7T fMRI study using a natural stimulus. Hum. Brain Mapp. 35, 213–226 (2014). 22965769 10.1002/hbm.22172 PMC6869627

[89] J. Kolasinski, T. R. Makin, S. Jbabdi, S. Clare, C. J. Stagg, H. Johansen-Berg, Investigating the stability of fine-grain digit somatotopy in individual human participants. J. Neurosci. 36, 1113–1127 (2016). 26818501 10.1523/JNEUROSCI.1742-15.2016 PMC4728720

[90] B. A. Wandell, S. O. Dumoulin, A. A. Brewer, Visual field maps in human cortex. Neuron 56, 366–383 (2007). 17964252 10.1016/j.neuron.2007.10.012

[91] F. Mancini, P. Haggard, G. D. Iannetti, M. R. Longo, M. I. Sereno, Fine-grained nociceptive maps in primary somatosensory cortex. J. Neurosci. 32, 17155–17162 (2012). 23197708 10.1523/JNEUROSCI.3059-12.2012 PMC3529201

[92] N. Zeharia, U. Hertz, T. Flash, A. Amedi, New whole-body sensory-motor gradients revealed using phase-locked analysis and verified using multivoxel pattern analysis and functional connectivity. J. Neurosci. 35, 2845–2859 (2015). 25698725 10.1523/JNEUROSCI.4246-14.2015 PMC6605596

[93] D. K. Deelchand, I. M. Adanyeguh, U. E. Emir, T.-M. Nguyen, R. Valabregue, P.-G. Henry, F. Mochel, G. Öz, Two-site reproducibility of cerebellar and brainstem neurochemical profiles with short-echo, single-voxel MRS at 3T. Magn. Reson. Med. 73, 1718–1725 (2015). 24948590 10.1002/mrm.25295 PMC4272339

[94] G. Oz, I. Tkac, Short-echo, single-shot, full-intensity proton magnetic resonance spectroscopy for neurochemical profiling at 4 T: Validation in the cerebellum and brainstem. Magn. Reson. Med. 65, 901–910 (2011). 21413056 10.1002/mrm.22708 PMC3827699

[95] O. Natt, V. Bezkorovaynyy, T. Michaelis, J. Frahm, Use of phased array coils for a determination of absolute metabolite concentrations. Magn. Reson. Med. 53, 3–8 (2005). 15690495 10.1002/mrm.20337

