# Supplementary material for: Malleability of the cortical hand map following a finger nerve block
Source: Sci Adv. 2022 Apr 22;8(16):eabk2393. doi: 10.1126/sciadv.abk2393 (PMC9032959; doi:10.1126/sciadv.abk2393)
Supplement: Supplementary file 1 — Supplementary Results Figs. S1 to S4 Tables S1 and S2 Supplementary Methods References [file sciadv.abk2393_sm.pdf]

Science Advances
AAAS

Supplementary Materials for

Malleability of the cortical hand map following a finger nerve block

Daan B. Wesselink*, Zeena-Britt Sanders, Laura R. Edmondson, Harriet Dempsey-Jones, Paulina Kieliba, Sanne Kikkert, Andreas C. Themistocleous, Uzay Emir, Jörn Diedrichsen, Hannes P. Saal, Tamar R. Makin

*Corresponding author. Email: dwesselink@gmail.com

Published 22 April 2022, *Sci. Adv.* **8**, eabk2393 (2022)
DOI: 10.1126/sciadv.abk2393

This PDF file includes:

Supplementary Results
Figs. S1 to S4
Tables S1 and S2
Supplementary Methods
References

Supplementary Results:

Univariate finger selectivity including D2

When D2 was included among non-target fingers, there was a significant decrease in selectivity across the finger clusters in the active task ($F_{(1,112)}$=5.00, p=.0273), but not in the passive task (F(1,112)=3.62, p=.059). The session x cluster interaction was not significant in either task ($F_{(3,112)}$=0.61, p=.607; and F(3,112)=0.41, p=.745, respectively).

Supplementary figures and tables:

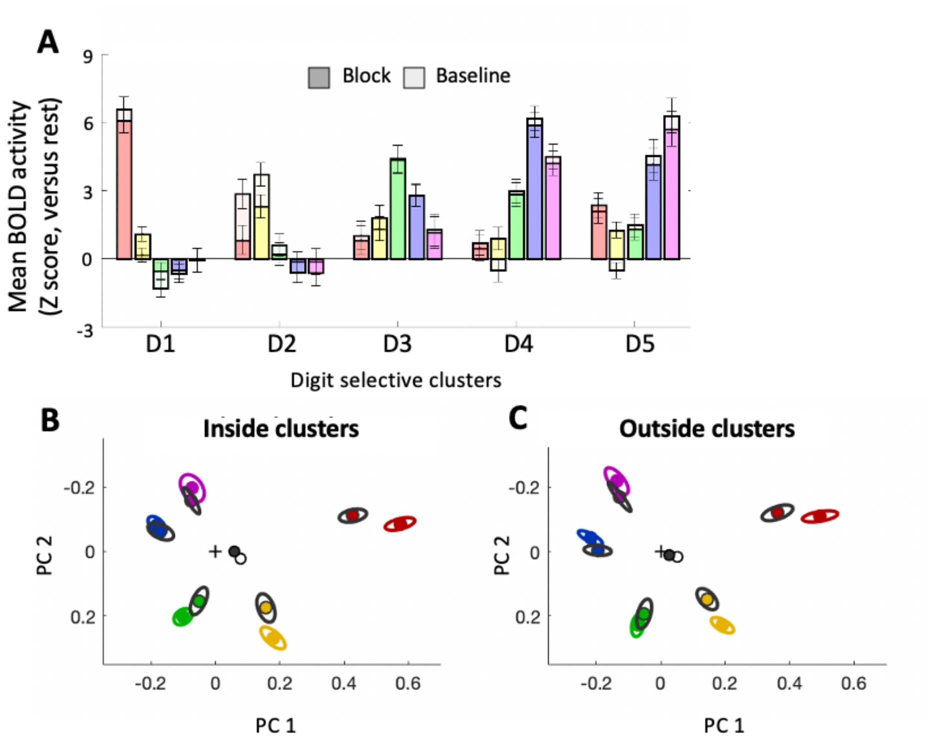

Figure S1: **Univariate and multivariate results from the active condition.** Related to Figures 2B and 3B-C. All other figure annotation is as detailed in the main figures. A) In the block session, active stimulation elicited positive activity in the deprived cluster C2 (μ=2.13; $t_{(14)}$=4.40, p=.001), but activity was significantly reduced compared to the baseline session ($t_{(14)}$=-2.22, p=.044). Mean activity in cluster C2 for the neighbouring fingers D1 and D3 was decreased in the block session ($t_{(14)}$=-2.51, p=.025). B-C) In line with the passive results, dissimilarity from rest during the active task shows a strong effect of session (B: $F_{(1,140)}$=10.7, p=.001; C: $F_{(1,140)}$=15.92, p<.001) and no session x finger interaction (B: $F_{(4,140)}$=0.21, p =.930; C: $F_{(4,140)}$=0,28, p=.891).

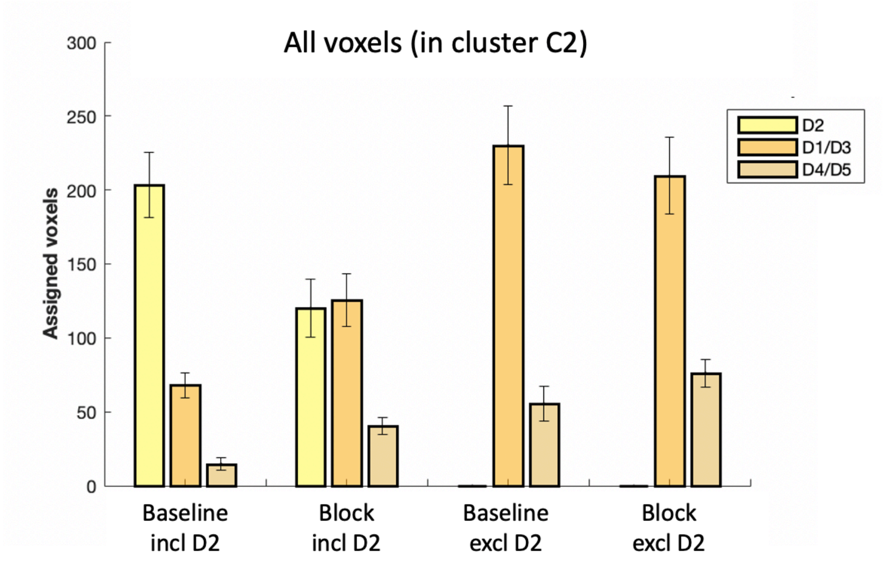

Figure S2: Quantification of 'remapping', using unthresholded voxels. Related to Figure 2D. All other figure annotations are as detailed in the main figure. As with the thresholded voxels, when D2 was ignored in both sessions (i.e. excluded from the winner-takes-all analysis), no significant difference in neighbouring fingers remapping was found between the baseline and block sessions (t(14)=1.61, p=0.131).

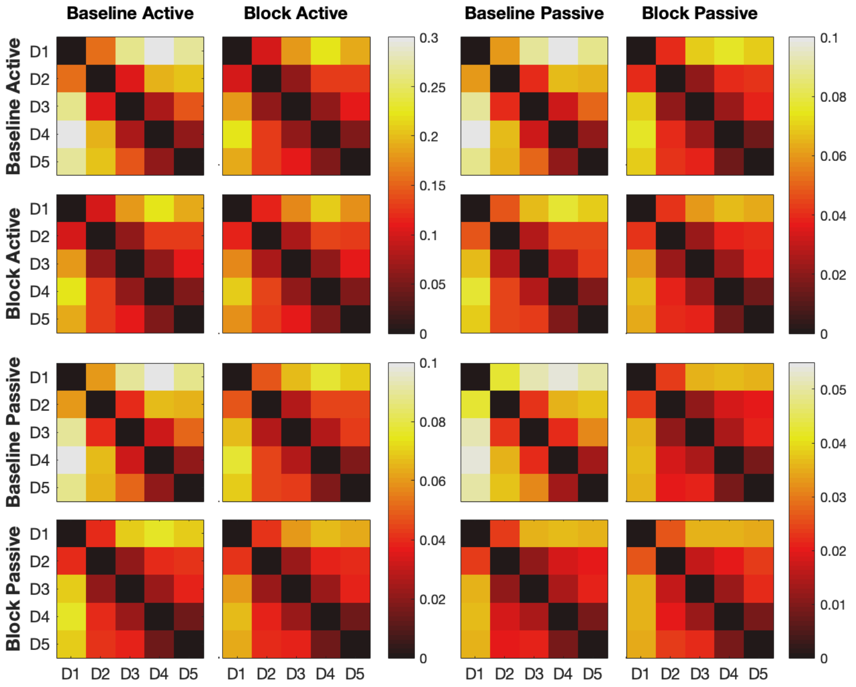

Figure S3: Representational dissimilarity matrix for all conditions, entire hand map. Related to Figure 3. Colours indicate Mahalanobis distance (arbitrary unit), please note scales vary between blocks. Each block compares finger activity patterns across conditions and sessions, assuming equal "rest state" (i.e. no activity) between sessions.

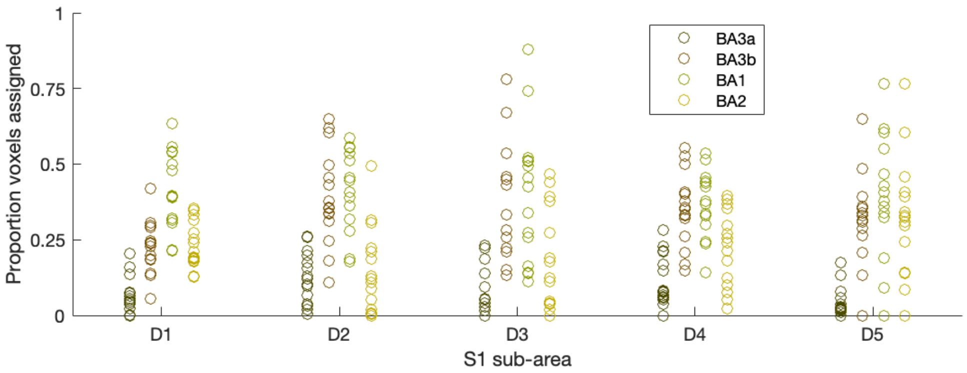

Figure S4: Distribution of finger clusters over S1 sub-areas. Related to Methods. Each dot depicts one participant. The y-axis indicates what proportion of each finger's finger cluster is assigned to each sub-area by the Freesurfer anatomical parcellation. This parcellation can assign each voxel multiple times (see Methods). Note both the high similarity between areas and the slight preference for areas 3b and 1.

Table S1. **Number of voxels per cluster.** For the RSA analysis of individual clusters (e.g. in Figure 3A), clusters with fewer than 50 voxels were excluded; these have been highlighted in cursive.

	C1	C2	C3	C4	C5	Total hand area
P1	481	155	*43*	324	211	12818
P2	456	229	268	391	197	7839
P3	468	151	388	380	124	7080
P4	284	290	252	358	54	6054
P5	610	213	219	598	241	5483
P6	694	425	221	*42*	368	6461
P7	136	336	276	260	66	5903
P8	351	96	162	225	*1*	5778
P9	92	176	50	154	253	6150
P10	719	270	65	292	302	8411
P11	374	398	304	609	235	7100
P12	194	187	109	284	212	5350
P13	230	76	*21*	331	249	5802
P14	368	140	54	416	127	5629
P15	771	235	222	172	83	5271

Table S2. GABA and Glutamate (GLu) values. GABA and Glu estimates of all individual subjects, together with their Cramér-Rao lower bounds (CRLBs), signal to noise ratio (SNR) and full width half maximum (FWHM). Note that subjects P1 and P12 were excluded from the analysis, due to unreliable GABA readings.

	Baseline				Block			
	GABA (CRLB)	Glu (CRLB)	SNR	FWHM	GABA (CRLB)	Glu (CRLB)	SNR	FWHM
P1	- (999)	6.75 (6)	34	0.03	1.90 (29)	8.24 (4)	43	0.03
P2	1.80 (41)	8.48 (5)	42	0.04	2.04 (23)	7.87 (4)	45	0.03
P3	1.77 (34)	7.43 (6)	38	0.03	2.04 (18)	5.45 (5)	54	0.03
P4	2.39 (17)	6.85 (4)	54	0.04	4.49 (10)	7.50 (3)	57	0.04
P5	1.71 (24)	7.48 (4)	51	0.03	1.53 (29)	7.10 (4)	48	0.03
P6	3.50 (16)	7.14 (4)	48	0.04	3.70 (15)	8.41 (4)	46	0.03
P7	4.07 (15)	8.61 (4)	47	0.04	2.49 (22)	7.81 (4)	45	0.04
P8	1.62 (29)	6.43 (4)	47	0.03	2.62 (18)	7.49 (4)	52	0.04
P9	2.12 (22)	7.53 (4)	48	0.03	2.50 (17)	6.32 (4)	48	0.03
P10	1.31 (34)	7.10 (4)	49	0.03	2.57 (26)	6.72 (6)	34	0.03
P11	0.78 (40)	5.99 (4)	57	0.03	1.47 (26)	6.77 (4)	55	0.03
P12	1.4 (30)	7.01 (4)	51	0.03	0.59 (55)	6.55 (4)	55	0.03

Supplementary Methods:

MRI acquisition & pre-processing

MRI acquisition

All MRI measurements were acquired using a Siemens 7 Tesla Magnetom scanner with a 32-channel head coil. Task fMRI data was acquired using a multiband EPI sequence with an acceleration factor of 2 (*82*,83). A limited field-of-view was used for fMRI acquisition, consisting of 56 slices of 1mm thick, centred over S1 with a 192x192mm in-plane FOV (TR 2000ms, TE 25ms, FA 85deg, GRAPPA 3). This resulted in a spatial resolution of 1mm isotropic. A whole brain anatomical T1-weighted image was also collected with 1mm isotropic spatial resolution (TR 2200ms, TE 2.82ms, FA 7deg, TI 1050ms).

1H MRS data was acquired and pre-processed as described in (*84*). A 2 x 1 x 1 cm voxel was placed manually over the hand knob in S1 using the collected T1-weighted anatomical scan. Three guidelines were followed to motivate correct placement (in order of importance): 1) the voxel avoided the dura mater, to prevent signal issues; 2) the voxel was placed posterior to the central sulcus, to limit the influence of M1; and 3) the voxel was placed as superior as possible to focus on the hand region. Due to data acquisition errors, data from three participants has been excluded from analysis. Two further participants were excluded from the analysis due to unreliable GABA quantification in one of the sessions (Cramér-Rao lower bounds higher than 50%). As such, the placement and resulting data quality was sufficient in both sessions to produce reliable spectra for 10 participants.

MRI pre-processing

All MRI data pre-processing and analysis was carried out using FMRIB Software Library (FSL; version 6.0) as well as Matlab scripts (version R2016a) which were developed in-house. Surface reconstruction was carried out using Freesurfer (*85*; www.freesurfer.net) and results from the task and travelling wave analysis were projected onto the cortical surface for visualisation purposes using Connectome Workbench software (www.humanconnectome.org).

Standard pre-processing steps were carried out for the fMRI data using FSL (*86*). FSL's Expert Analysis Tool (FEAT) was used to carry out motion correction (using MCFLIRT; *8*7, brain extraction (BET; *88)*, spatial smoothing of all fMRI data using a 1mm full width at half maximum (FWHM) Gaussian kernel and high pass filtering using a cut-off of 100s. The output from the MCFLIRT analysis was visually inspected for excessive motion (defined as >1mm absolute mean displacement). No participants had an absolute mean displacement greater than 1mm.

Image registration

For each participant, a mid-space was calculated between the four active and four passive runs, i.e. the average space in which the images are minimally reoriented. Each scan was then aligned to this session mid-space using FMRIB's Linear Image Registration Tool (FLIRT; 6 DOF; *86*, *89*). The two runs of the functional localiser were also registered to the mid-space of the baseline session (but first to each other). The structural scans from both sessions were also combined by finding a mid-space. The functional mid-spaces from both sessions were registered to this anatomical mid-space using FLIRT together with manual

adjustments to ensure an accurate co-registration of the central sulcus (specifically, the "hand knob"). Once co-registration was satisfactory, all functional scans across both sessions were aligned to this anatomical mid-space.

MRI tasks

Passive task

In the passive stimulation task, we asked the participants to rest their right hand in a comfortable, supine position on a foam cushion. An experimenter stimulated each finger by tapping a plastic probe against the distal pad of the finger. Such manual stimulation is commonly used in somatosensory studies to limit "contamination" from the motor system. Although manual stimulation may be less localised than other (e.g. pneumatic) methods, it has previously shown to robustly activate finger maps (*32, 90*. Also, even highly precise (1 mm) vibrational stimulation fails to remain localised due to indirect stimulation (i.e. skin ripples discussed earlier) (see *7*; http://movie-usa.glencoesoftware.com/video/10.1073/pnas.1704856114/video-1).

The experimenter was instructed through headphones. Any slight variations produced by the experimenter were meant to account for variations in the active task (below) and were not likely to differ between the baseline and block sessions. During the passive task, participants were shown dots flashing synchronously with the tactile stimulation to indicate when and where a touch occurred. This way, the passive and active task both featured task-related visual input.

To further promote engagement across the duration of the task, double taps were administered sporadically (one double tap per finger condition in each run). Participants were asked to press a button with their left hand when they felt a double tap. Participants correctly identified these catch trials in 93.3% of the cases in the baseline session (excluding D2 trials). This percentage was 94.2% in the block session. There was no significant difference in double tap detection between (non-blocked) fingers ($F(3,112)=1.97$, $p=.123$) or between sessions ($F(1,112)=0.10$, $p=.748$). Detection of D2 double taps was impaired in the block session (66.7%) compared to baseline (98.3%, $t(14)=3.68$, $p=.002$). We note that this task was designed to maintain the participants' engagement throughout the scan and was not suitable as a tactile perceptual test. Most notably, we cannot guarantee participants did not use alternative cues (e.g., based on peripheral vision; skin ripples on neighbouring skin) to detect double taps. More rigorous tests of perception suggest an effective attenuation of input (see Fig 1D and Methods - *Tactile perceptual analysis*). Overall, the high detection rate for all unblocked fingers suggests the participants remained attentive throughout the passive task.

Active task

The active task was a visually cued (motor) task. In an intact sensorimotor system, movement recruits a combination of peripheral receptors, encoding a range of somatosensory modalities (e.g., surface and deeper mechanoreceptors; proprioceptors), as well as efferent information from the motor system. Using an active task, we have previously shown high consistency of S1 finger topography across multiple scanning sessions (*91*, see also *9* for validation using RSA). Participants were presented with five vertical bars, corresponding to the five fingers, shown on a visual display projected into the scanner bore. To cue

the participant which finger should be moved, the bar corresponding to this finger changed (i.e., by flashing in a different colour).

The participants performed the tasks well. The instructed finger produced the strongest press force in 94.6% of the trials (92.2% in the worst participant). Consequently, there was a clear difference in average force output between the instructed and non-instructed fingers: In the baseline session, 1.44 N (+/- 0.09 SEM) for the instructed finger and 0.27 N (+/- 0.03) for non-instructed fingers; and in the block session, 1.39 N (+/- 0.07) and 0.24 N (+/- 0.03) respectively. There was no difference in force output between sessions ($F(1,140)=0.28$, $p=.596$). This was also the case when only D2 output was compared ($t(14)=1.31$, $p=.211$), suggesting any differences between sessions are not due to impaired motor performance.

Finger-selective cluster localiser

We also conducted a functional localiser before the active task in the baseline session to independently identify finger-selective regions of interest (here termed clusters C1-C5). This localiser was also organised into finger-specific blocks, but with a set inter-finger sequence design ('travelling wave design'; *28*, *91-94*). This approach is particularly useful for identifying voxels that show an enhanced response to one finger compared to all other fingers and has previously been used to identify S1 finger somatotopy (e.g., *91*).

Two runs were acquired, with a reversed order from each other, each consisting of 108 volumes, covering 5 cycles around the hand. The travelling wave protocol involves a set finger cycle. Participants used the same keyboard and visual display as in the active task. Two separate runs, with a reverse order of conditions (i.e., a forward and a backward cycle), were used to overcome potential order-related biases due to the sluggish haemodynamic response. In the forward run, the order of finger blocks cycled from finger 1 to finger 5 (D1-D2-D3-D4-D5) whereas a reverse order of finger blocks was used in the backward run (D5-D4-D3-D2-D1). In each run, the cycle was repeated five times with no rest periods in between. During the cycles, each finger was moved 8 times (at 1Hz) before the instructions for the next finger were shown. As in the active task, the finger to be used in the upcoming block was visually cued at the start of each block, followed by 8 finger presses of that same finger.

Resting state scan

Participants were instructed to keep their eyes open and gaze at a fixation cross. Otherwise, they were instructed to let their mind wander and not think of anything in particular. 150 volumes were acquired.

Magnetic resonance spectroscopy

1H MRS was acquired using 2x1x1 voxel placed manually over the hand knob in S1, using the collected T1-weighted anatomical scan. Three guidelines were followed to motivate correct placement (in order of importance): 1) the voxel avoided the dura matter, to prevent signal issues; 2) the voxel was placed posterior to the central sulcus, to limit the influence of M1; and 3) the voxel was placed as far superior as possible, to focus on the hand region.

Spectra were measured with a semi-adiabatic localization by adiabatic selective refocusing (semi-LASER)

sequence (TE=36ms, TR = 5s, 64 averages) with variable power RF pulses with optimized relaxation delays (VAPOR), water suppression and outer volume saturation (*95*,*96*). Unsuppressed water spectra acquired from the same volume of interest were used to remove residual eddy current effects and to reconstruct the phased array spectra *(97*).

MRS metabolites were quantified using LCmodel. The model spectra of alanine (Ala), aspartate (Asp), ascorbate/vitamin C (Asc), glycerophosphocholine (GPC), phosphocholine (PC), creatine (Cr), phosphocreatine (PCr), GABA, glucose, glutamine (Gln), glutamate (Glu), glutathione, lactate (Lac), *myo*-Inositol (*myo*-Ins), NAA, N-acetylaspartylglutamate, phosphoethanolamine (PE), scyllo-Inositol (*scyllo*-Ins) and taurine were generated based on previously reported chemical shifts and coupling constants by the GAMMA/PyGAMMA simulation library of VeSPA (Versatile Simulation, Pulses and Analysis) according to a density matrix formalism. Simulations were performed with the same RF pulses and sequence timings as those on the 7T system in use. Resonances were assigned according to their known 1H chemical shift along the spectrum (x-axis, in parts per million). The T2 relaxation of tissue water content (80 ms; *95*) was taken into account in the LCmodel fitting. Absolute neurochemical concentrations of GABA and Glutamate were extracted from the spectra of the greater S1 hand area while correcting for voxel tissue content (*84*). Metabolites quantified with Cramér-Rao lower bounds higher than 50% (estimated error of the metabolite quantification) were classified as not detected. The Glutamate/GABA ratios for all participants were compared across sessions using a paired t-test.

REFERENCES AND NOTES

1. J. H. Kaas, R. J. Nelson, M. Sur, C.-S. Lin, M. M. Merzenich, Multiple representations of the body within the primary somatosensory cortex of primates. *Science* **204**, 521–523 (1979).

2. R. M. Sanchez-Panchuelo, S. Francis, R. Bowtell, D. Schluppeck, Mapping human somatosensory cortex in individual subjects with 7T functional MRI. *J. Neurophysiol.* **103**, 2544–2556 (2010).

3. P. H. Thakur, P. J. Fitzgerald, S. S. Hsiao, Second-order receptive fields reveal multidigit interactions in area 3b of the macaque monkey. *J. Neurophysiol.* **108**, 243–262 (2012).

4. T. M. McKenna, B. L. Whitsel, D. A. Dreyer, Anterior parietal cortical topographic organization in macaque monkey: A reevaluation. *J. Neurophysiol.* **48**, 289–317 (1982).

5. M. Tommerdahl, O. V. Favorov, B. L. Whitsel, Dynamic representations of the somatosensory cortex. *Neurosci. Biobehav. Rev.* **34**, 160–170 (2010).

6. L. R. Manfredi, A. T. Baker, D. O. Elias, J. F. Dammann, M. C. Zielinski, V. S. Polashock, S. J. Bensmaia, The effect of surface wave propagation on neural responses to vibration in primate glabrous skin. *PLOS ONE* **7**, e31203 (2012).

7. H. P. Saal, B. P. Delhaye, B. C. Rayhaun, S. J. Bensmaia, Simulating tactile signals from the whole hand with millisecond precision. *Proc. Natl. Acad. Sci. U.S.A.* **114**, E5693–E5702 (2017).

8. Y. Shao, V. Hayward, Y. Visell, Compression of dynamic tactile information in the human hand. *Sci. Adv.* **6**, eaaz1158 (2020).

9. N. Ejaz, M. Hamada, J. Diedrichsen, Hand use predicts the structure of representations in sensorimotor cortex. *Nat. Neurosci.* **18**, 1034–1040 (2015).

10. H. Dempsey-Jones, V. Harrar, J. Oliver, H. Johansen-Berg, C. Spence, T. R. Makin, Transfer of tactile perceptual learning to untrained neighbouring fingers reflects natural use relationships. *J. Neurophysiol.* **115**, 1088–1097 (2016).

11. E. Kuehn, B. Pleger, Encoding schemes in somatosensation: From micro- to meta-topography. *Neuroimage* **223**, 117255 (2020).

12. D. Muret, V. Root, P. Kieliba, D. Clode, T. R. Makin, Beyond body maps: Information content of specific body parts is distributed across the somatosensory homunculus. *Cell Rep.* **38**, 110523 (2022).

13. D. V. Buonomano, M. M. Merzenich, Cortical plasticity: From synapses to maps. *Annu. Rev. Neurosci.* **21**, 149–186 (1998).

14. M. M. Merzenich, J. H. Kaas, J. T. Wall, M. Sur, R. J. Nelson, D. J. Felleman, Progression of change following median nerve section in the cortical representation of the hand in areas 3b and 1 in adult owl and squirrel monkeys. *Neuroscience* **10**, 639–665 (1983).

15. B. M. Faggin, K. T. Nguyen, M. A. Nicolelis, Immediate and simultaneous sensory reorganization at cortical and subcortical levels of the somatosensory system. *Proc. Natl. Acad. Sci. U.S.A.* **94**, 9428–9433 (1997).

16. T. P. Pons, P. E. Garraghty, A. K. Ommaya, J. H. Kaas, E. Taub, M. Mishkin, Massive cortical reorganization after sensory deafferentation in adult macaques. *Science* **252**, 1857–1860 (1991).

17. J. D. Churchill, N. Muja, W. A. Myers, J. Besheer, P. E. Garraghty, Somatotopic consolidation: A third phase of reorganization after peripheral nerve injury in adult squirrel monkeys. *Exp. Brain Res.* **118**, 189–196 (1998).

18. M. M. Merzenich, R. J. Nelson, M. P. Stryker, M. S. Cynader, A. Schoppmann, J. M. Zook, Somatosensory cortical map changes following digit amputation in adult monkeys. *J Comp Neurol* **224**, 591–605 (1984).

19. M. M. Merzenich, J.H. Kaas, J. Wall, R.J. Nelson, M. Sur, D. Felleman, Topographic reorganization of somatosensory cortical areas 3b and 1 in adult monkeys following restricted deafferentation. *Neuroscience* **8**, 33–55 (1983).

20. D. D. Rasmusson, Reorganization of raccoon somatosensory cortex following removal of the fifth digit. *J. Comp. Neurol.* **205**, 313–326 (1982).

21. J. H. Kaas, Plasticity of sensory and motor maps in adult mammals. *Annu. Rev. Neurosci.* **14**, 137–167 (1991).

22. N. M. Weinberger, Dynamic regulation of receptive fields and maps in the adult sensory cortex. *Annu. Rev. Neurosci.* **18**, 129–158 (1995).

23. D. E. Feldman, M. Brecht, Map plasticity in somatosensory cortex. *Science* **310**, 810–815 (2005).

24. M. B. Calford, R. Tweedale, Immediate expansion of receptive fields of neurons in area 3b of macaque monkeys after digit denervation. *Somatosens. Mot. Res.* **8**, 249–260 (1991).

25. T. R. Makin, S. J. Bensmaia, Stability of sensory topographies in adult cortex. *Trends Cogn. Sci.* **21**, 195–204 (2017).

26. S. N. Flesher, J. E. Downey, J. M. Weiss, C. L. Hughes, A. J. Herrera, E. C. Tyler-Kabara, M. L. Boninger, J. L. Collinger, R. A. Gaunt, A brain-computer interface that evokes tactile sensations improves robotic arm control. *Science* **372**, 831–836 (2021).

27. S. Kikkert, D. Pfyffer, M. Verling, P. Freund, N. Wenderoth, Finger somatotopy is preserved after tetraplegia but deteriorates over time. *eLife* **10**, e67713 (2021).

28. S. Kikkert, J. Kolasinski, S. Jbabdi, I. Tracey, C. F. Beckmann, H. J. Berg, T. R. Makin, Revealing the neural fingerprints of a missing hand. *Elife* **5**, e15292 (2016).

29. D. B. Wesselink, F. M. Z. van den Heiligenberg, N. Ejaz, H. Dempsey-Jones, L. Cardinali, A. Tarall-Jozwiak, J. Diedrichsen, T. R. Makin, Obtaining and maintaining cortical hand representation as evidenced from acquired and congenital handlessness. *eLife* **8**, e37227 (2019).

30. M. Bruurmijn, I. P. L. Pereboom, M. J. Vansteensel, M. A. H. Raemaekers, N. F. Ramsey, Preservation of hand movement representation in the sensorimotor areas of amputees. *Brain* **140**, 3166–3178 (2017).

31. A. Serino, M. Akselrod, R. Salomon, R. Martuzzi, M. L. Blefari, E. Canzoneri, G. Rognini, W. van der Zwaag, M. Iakova, F. Luthi, A. Amoresano, T. Kuiken, O. Blanke, Upper limb cortical maps in amputees with targeted muscle and sensory reinnervation. *Brain* **140**, 2993–3011 (2017).

32. Z.-B. Sanders, D. B. Wesselink, H. Dempsey-Jones, T. R. Makin, Similar somatotopy for active and passive digit representation in primary somatosensory cortex. bioRxiv 754648 [**Preprint**]. 5 September 2019. https://doi.org/10.1101/754648.

33. S. Raymond, A. Gissen, Mechanisms of Differential Nerve Block, in *Local Anesthetics*, G. R. Strichartz, Ed. (Springer, 1987), pp. 95–164.

34. C. Mehring, M. Akselrod, L. Bashford, M. Mace, H. Choi, M. Blüher, A.-S. Buschhoff, T. Pistohl, R. Salomon, A. Cheah, O. Blanke, A. Serino, E. Burdet, Augmented manipulation ability in humans with six-fingered hands. *Nat. Commun.* **10**, 2401 (2019).

35. P. Kieliba, D. Clode, R. O. Maimon-Mor, T. R. Makin, Robotic hand augmentation drives changes in neural body representation. *Sci. Robot.* **6**, eabd7935 (2021).

36. C. J. Stagg, S. Bestmann, A. O. Constantinescu, L. M. Moreno, C. Allman, R. Mekle, M. Woolrich, J. Near, H. Johansen-Berg, J. C. Rothwell, Relationship between physiological measures of excitability and levels of glutamate and GABA in the human motor cortex. *J. Physiol.* **589**, 5845–5855 (2011).

37. H. Flor, T. Elbert, S. Knecht, C. Wienbruch, C. Pantev, N. Birbaumers, W. Larbig, E. Taub, Phantom-limb pain as a perceptual correlate of cortical reorganization following arm amputation. *Nature* **375**, 482–484 (1995).

38. L. M. Chen, H. X. Qi, J. H. Kaas, Dynamic reorganization of digit representations in somatosensory cortex of nonhuman primates after spinal cord injury. *J. Neurosci.* **32**, 14649–14663 (2012).

39. H. Dempsey-Jones, A. C. Themistocleous, D. Carone, T. W. C. Ng, V. Harrar, T. R. Makin, Blocking tactile input to one finger using anaesthetic enhances touch perception and learning in other fingers. *J. Exp. Psychol.* **148**, 713–727 (2019).

40. J. Xing, G. L. Gerstein, Simulation of dynamic receptive fields in primary visual cortex. *Vision Res.* **34**, 1901–1911 (1994).

41. K. Ogawa, K. Mitsui, F. Imai, S. Nishida, Long-term training-dependent representation of individual finger movements in the primary motor cortex. *Neuroimage* **202**, 116051 (2019).

42. B. Godde, F. Spengler, H. R. Dinse, Associative pairing of tactile stimulation induces somatosensory cortical reorganization in rats and humans. *Neuroreport* **8**, 281–285 (1996).

43. N. Kambi, P. Halder, R. Rajan, V. Arora, P. Chand, M. Arora, N. Jain, Large-scale reorganization of the somatosensory cortex following spinal cord injuries is due to brainstem plasticity. *Nat. Commun.* **5**, 3602 (2014).

44. J. A. Pruszynski, R. S. Johansson, Edge-orientation processing in first-order tactile neurons. *Nat. Neurosci.* **17**, 1404–1409 (2014).

45. H. P. Saal, M. A. Harvey, S. J. Bensmaia, Rate and timing of cortical responses driven by separate sensory channels. *eLife* **4**, e10450 (2015).

46. J. N. Ingram, K. P. Kording, I. S. Howard, D. M. Wolpert, The statistics of natural hand movements. *Exp. Brain Res.* **188**, 223–236 (2008).

47. R. M. Sanchez Panchuelo, R. Ackerley, P. M. Glover, R. W. Bowtell, J. Wessberg, S. T. Francis, F. McGlone, Mapping quantal touch using 7 Tesla functional magnetic resonance imaging and single-unit intraneural microstimulation. *eLife* **5**, e12812 (2016).

48. G. G. Turrigiano, S. B. Nelson, Hebb and homeostasis in neuronal plasticity. *Curr. Opin. Neurobiol.* **10**, 358–364 (2000).

49. F. Zenke, W. Gerstner, S. Ganguli, The temporal paradox of Hebbian learning and homeostatic plasticity. *Curr. Opin. Neurobiol.* **43**, 166–176 (2017).

50. E. Castaldi, C. Lunghi, M. C. Morrone, Neuroplasticity in adult human visual cortex. *Neurosci. Biobehav. Rev.* **112**, 542–552 (2020).

51. B. Nystrom, K. E. Hagbarth, Microelectrode recordings from transected nerves in amputees with phantom limb pain. *Neurosci. Lett.* **27**, 211–216 (1981).

52. S. Lee, G. E. Carvell, D. J. Simons, Motor modulation of afferent somatosensory circuits. *Nat. Neurosci.* **11**, 1430–1438 (2008).

53. R. A. Adams, S. Shipp, K. J. Friston, Predictions not commands: Active inference in the motor system. *Brain Struct. Funct.* **218**, 611–643 (2013).

54. K. D. Davis, Z. H. Kiss, L. Luo, R. R. Tasker, A. M. Lozano, J. O. Dostrovsky, Phantom sensations generated by thalamic microstimulation. *Nature* **391**, 385–387 (1998).

55. E. Kuehn, P. Haggard, A. Villringer, B. Pleger, M. I. Sereno, Visually-driven maps in area 3b. *J. Neurosci.* **38**, 1295–1310 (2018).

56. M. Jafari, T. Aflalo, S. Chivukula, S. S. Kellis, M. A. Salas, S. L. Norman, K. Pejsa, C. Y. Liu, R. A. Andersen, The human primary somatosensory cortex encodes imagined movement in the absence of sensory information. *Commun. Biol.* **3**, 757 (2020).

57. A. M. Puckett, S. Bollmann, M. Barth, R. Cunnington, Measuring the effects of attention to individual fingertips in somatosensory cortex using ultra-high field (7T) fMRI. *Neuroimage* **161**, 179–187 (2017).

58. F. W. Smith, L. Muckli, Nonstimulated early visual areas carry information about surrounding context. *Proc. Natl. Acad. Sci. U.S.A.* **107**, 20099–20103 (2010).

59. F. P. de Lange, M. Heilbron, P. Kok, How do expectations shape perception? *Trends Cogn. Sci.* **22**, 764–779 (2018).

60. J. L. Reed, P. Pouget, H.-X. Qi, Z. Zhou, M. R. Bernard, M. J. Burish, J. Haitas, A B Bonds, J. H. Kaas, Widespread spatial integration in primary somatosensory cortex. *Proc. Natl. Acad. Sci. U.S.A.* **105**, 10233–10237 (2008).

61. D. Muret, T. R. Makin, The homeostatic homunculus: Rethinking deprivation-triggered reorganisation. *Curr. Opin. Neurobiol.* **67**, 115–122 (2021).

62. T. R. Makin, H. Flor, Brain (re)organisation following amputation: Implications for phantom limb pain. *Neuroimage* **218**, 116943 (2020).

63. D. D. Rasmusson, D. M. Nance, Non-overlapping thalamocortical projections for separate forepaw digits before and after cortical reorganization in the raccoon. *Brain Res. Bull.* **16**, 399–406 (1986).

64. D. E. Feldman, Synaptic mechanisms for plasticity in neocortex. *Annu. Rev. Neurosci.* **32**, 33–55 (2009).

65. B. G. Turnbull, D. D. Rasmusson, Acute effects of total or partial digit denervation on raccoon somatosensory cortex. *Somatosens. Mot. Res.* **7**, 365–389 (1990).

66. R. C. Kolarik, S. K. Rasey, J. T. Wall, The consistency, extent, and locations of early-onset changes in cortical nerve dominance aggregates following injury of nerves to primate hands. *J. Neurosci.* **14**, 4269–4288 (1994).

67. F. Panetsos, A. Nunez, C. Avendano, Local anaesthesia induces immediate receptive field changes in nucleus gracilis and cortex. *Neuroreport* **7**, 150–152 (1995).

68. A. Bjorkman, A. Weibull, B. Rosen, J. Svensson, G. Lundborg, Rapid cortical reorganisation and improved sensitivity of the hand following cutaneous anaesthesia of the forearm. *Eur. J. Neurosci.* **29**, 837–844 (2009).

69. T. Weiss, W. H. Miltner, J. Liepert, W. Meissner, E. Taub, Rapid functional plasticity in the primary somatomotor cortex and perceptual changes after nerve block. *Eur. J. Neurosci.* **20**, 3413–3423 (2004).

70. K. J. Werhahn, J. Mortensen, R. W. Van Boven, K. E. Zeuner, L. G. Cohen, Enhanced tactile spatial acuity and cortical processing during acute hand deafferentation. *Nat. Neurosci.* **5**, 936–938 (2002).

71. M. B. Calford, Dynamic representational plasticity in sensory cortex. *Neuroscience* **111**, 709–738 (2002).

72. M. Sur, P. E. Garraghty, C. J. Bruce, Somatosensory cortex in macaque monkeys: Laminar differences in receptive field size in areas 3b and 1. *Brain Res.* **342**, 391–395 (1985).

73. J. Goense, H. Merkle, N. K. Logothetis, High-resolution fMRI reveals laminar differences in neurovascular coupling between positive and negative BOLD responses. *Neuron* **76**, 629–639 (2012).

74. A. Karl, N. Birbaumer, W. Lutzenberger, L. G. Cohen, H. Flor, Reorganization of motor and somatosensory cortex in upper extremity amputees with phantom limb pain. *J. Neurosci.* **21**, 3609–3618 (2001).

75. A. Pascual-Leone, M. Peris, J. M. Tormos, A. P. Pascual, M. D. Catalá, Reorganization of human cortical motor output maps following traumatic forearm amputation. *Neuroreport* **7**, 2068–2070 (1996).

76. R. Mukamel, H. Gelbard, A. Arieli, U. Hasson, I. Fried, R. Malach, Coupling between neuronal firing, field potentials, and FMRI in human auditory cortex. *Science* **309**, 951–954 (2005).

77. N. Kriegeskorte, M. Mur, P. Bandettini, Representational similarity analysis—Connecting the branches of systems neuroscience. *Front. Syst. Neurosci.* **2**, 4 (2008).

78. A. Walther, H. Nili, N. Ejaz, A. Alink, N. Kriegeskorte, J. Diedrichsen, Reliability of dissimilarity measures for multi-voxel pattern analysis. *Neuroimage* **137**, 188–200 (2016).

79. R. Wetzels, D. Matzke, M. D. Lee, J. N. Rouder, G. J. Iverson, E.J. Wagenmakers, Statistical evidence in experimental psychology: An empirical comparison using 855 t tests. *Perspect. Psychol. Sci.* **6**, 291–298 (2011).

80. S. Moeller, E. Yacoub, C.A. Olman, E. Auerbach, J. Strupp, N. Harel, K. Uğurbil, Multiband multislice GE-EPI at 7 tesla, with 16-fold acceleration using partial parallel imaging with application to high spatial and temporal whole-brain fMRI. *Magn. Reson. Med.* **63**, 1144–1153 (2010).

81. K. Ugurbil, J. Xu, E. J. Auerbach, S. Moeller, A. T. Vu, J. M. Duarte-Carvajalino, C. Lenglet, X. Wu, S. Schmitter, Pierre Francois Van de Moortele, J. Strupp, G. Sapiro, F. De Martino, D. Wang, N. Harel, M. Garwood, L. Chen, D. A. Feinberg, S. M. Smith, K. L. Miller, S. N. Sotiropoulos, S. Jbabdi, J. L. R. Andersson, T. E. J. Behrens, M. F. Glasser, D. C. Van Essen, E. Yacoub; WU-Minn HCP Consortium, Pushing spatial and temporal resolution for functional and diffusion MRI in the Human Connectome Project. *Neuroimage* **80**, 80–104 (2013).

82. C. Lunghi, U. E. Emir, M. C. Morrone, H. Bridge, Short-term monocular deprivation alters GABA in the adult human visual cortex. *Curr. Biol.* **25**, 1496–1501 (2015).

83. A. M. Dale, B. Fischl, M. I. Sereno, Cortical surface-based analysis. I. Segmentation and surface reconstruction. *Neuroimage* **9**, 179–194 (1999).

84. M. Jenkinson, C. F. Beckmann, T. E. J. Behrens, M. W. Woolrich, S. M. Smith, FSL. *Neuroimage* **62**, 782–790 (2012).

85. M. Jenkinson, P. Bannister, M. Brady, S. Smith, Improved optimization for the robust and accurate linear registration and motion correction of brain images. *Neuroimage* **17**, 825–841 (2002).

86. S. M. Smith, Fast robust automated brain extraction. *Hum. Brain Mapp.* **17**, 143–155 (2002).

87. M. Jenkinson, S. Smith, A global optimisation method for robust affine registration of brain images. *Med. Image Anal.* **5**, 143–156 (2001).

88. R. Martuzzi, W. van der Zwaag, J. Farthouat, R. Gruetter, O. Blanke, Human finger somatotopy in areas 3b, 1, and 2: A 7T fMRI study using a natural stimulus. *Hum. Brain Mapp.* **35**, 213–226 (2014).

89. J. Kolasinski, T. R. Makin, S. Jbabdi, S. Clare, C. J. Stagg, H. Johansen-Berg, Investigating the stability of fine-grain digit somatotopy in individual human participants. *J. Neurosci.* **36**, 1113–1127 (2016).

90. B. A. Wandell, S. O. Dumoulin, A. A. Brewer, Visual field maps in human cortex. *Neuron* **56**, 366–383 (2007).

91. F. Mancini, P. Haggard, G. D. Iannetti, M. R. Longo, M. I. Sereno, Fine-grained nociceptive maps in primary somatosensory cortex. *J. Neurosci.* **32**, 17155–17162 (2012).

92. N. Zeharia, U. Hertz, T. Flash, A. Amedi, New whole-body sensory-motor gradients revealed using phase-locked analysis and verified using multivoxel pattern analysis and functional connectivity. *J. Neurosci.* **35**, 2845–2859 (2015).

93. D. K. Deelchand, I. M. Adanyeguh, U. E. Emir, T.-M. Nguyen, R. Valabregue, P.-G. Henry, F. Mochel, G. Öz, Two-site reproducibility of cerebellar and brainstem neurochemical profiles with short-echo, single-voxel MRS at 3T. *Magn. Reson. Med.* **73**, 1718–1725 (2015).

94. G. Oz, I. Tkac, Short-echo, single-shot, full-intensity proton magnetic resonance spectroscopy for neurochemical profiling at 4 T: Validation in the cerebellum and brainstem. *Magn. Reson. Med.* **65**, 901–910 (2011).

95. O. Natt, V. Bezkorovaynyy, T. Michaelis, J. Frahm, Use of phased array coils for a determination of absolute metabolite concentrations. *Magn. Reson. Med.* **53**, 3–8 (2005).
